# Effects of Xylanase and Protease Supplementation on Growth Performance, Meat Quality, Gut Health, Cecal Fermentation, and Bone Traits in Broiler Chickens

**DOI:** 10.3390/ani16030465

**Published:** 2026-02-02

**Authors:** Esin Ebru Onbaşılar, Sakine Yalçın, Barış Batur, Suzan Yalçın, İhsan Berat Kılıçlı, Caner Bakıcı, Buket Bakır, Yeliz Kaya Kartal, Tevhide Sel

**Affiliations:** 1 Department of Animal Husbandry, Faculty of Veterinary Medicine, Ankara University, 06110 Ankara, Türkiye; 2 Animal Nutrition Science Association, 06420 Ankara, Türkiye; 3 Department of Anatomy, Faculty of Veterinary Medicine, Ankara University, 06110 Ankara, Türkiye; barisbaturr@gmail.com (B.B.); vetcanerbakici@gmail.com (C.B.); 4 The Graduate School of Health Sciences, Ankara University, 06110 Ankara, Türkiye; 5 Department of Food Hygiene and Technology, Faculty of Veterinary Medicine, Selçuk University, 42003 Konya, Türkiye; syalcin@selcuk.edu.tr; 6 Faculty of Veterinary Medicine, Ankara University, 06110 Ankara, Türkiye; kilicli571@gmail.com; 7 Department of Histology and Embryology, Faculty of Veterinary Medicine, Tekirdağ Namık Kemal University, 59030 Tekirdağ, Türkiye; buketbakir@nku.edu.tr; 8 Department of Biochemistry, Faculty of Veterinary Medicine, Ankara University, 06110 Ankara, Türkiye; ylzkaya@ankara.edu.tr (Y.K.K.); sel@veterinary.ankara.edu.tr (T.S.)

**Keywords:** broiler chickens, xylanase, protease, bone morphometry, intestinal morphology, growth performance

## Abstract

This study investigated the effects of dietary xylanase and protease supplementation, applied individually or in combination, on growth performance, intestinal characteristics, gut fermentation, meat quality, and skeletal traits in broiler chickens. A total of 540-day-old male broiler chicks were allocated to six experimental groups and fed a control corn-soybean meal-based diet or diets supplemented with xylanase, protease, or a xylanase–protease combination. Enzyme supplementation significantly improved body weight gain and feed efficiency, particularly between days 22 and 42, and reduced intestinal digesta viscosity. Improvements in gut morphology were reflected by increased villus height and villus-to-crypt ratios, accompanied by higher cecal total volatile fatty acid concentrations, increased *Lactobacillus* populations, and reduced coliform counts. In contrast, breast meat physicochemical composition and antioxidant status were not affected by dietary treatments. Skeletal development was positively influenced, with improvements observed in selected morphometric and structural bone traits. Overall, dietary xylanase and protease supplementation enhanced broiler performance and skeletal development primarily through improved digestive efficiency and favorable modulation of gut morphology, microbial ecology, and intestinal fermentation, without adverse effects on meat quality.

## Simple Summary

In modern broiler production, rapid growth may challenge skeletal development due to limitations in nutrient utilization rather than inadequate mineral supply. This study evaluated the effects of dietary xylanase and protease supplementation, applied individually or in combination, on growth performance, intestinal characteristics, meat quality, gut health, and bone development in broiler chickens. Enzyme supplementation mainly enhanced growth performance during the 22–42-day period, which was associated with reduced intestinal viscosity and improved digestive efficiency. Breast meat composition and antioxidant status were not affected by the dietary treatments. Despite identical dietary calcium and phosphorus levels, enzyme-supplemented broilers exhibited improvements in bone development, including increased bone size, weight, and structural robustness, particularly in the *femur* and lower leg bones. These responses likely reflect enhanced nutrient utilization rather than changes in mineral intake. Overall, dietary xylanase and protease supplementation represents a nutritional strategy to support growth performance, gut function, and skeletal integrity in fast-growing broiler chickens.

## 1. Introduction

The continuous genetic improvement of modern broiler chickens has led to substantial gains in growth rate, feed efficiency, and breast meat yield over recent decades [1,2]. While these advances have markedly increased production efficiency, they have also intensified challenges related to nutrient utilization, gastrointestinal function, and skeletal development, particularly in fast-growing genotypes [3,4]. Rapid body weight (BW) accretion often outpaces skeletal mineralization, increasing the incidence of leg disorders and compromised welfare [1,5,6,7]. Consequently, nutritional strategies that support digestive efficiency, performance, and skeletal integrity are of growing importance.

Corn–soybean meal (CSBM)-based diets represent the predominant feeding strategy for broilers due to their high energy density and favorable amino acid profile, and consistent availability. However, despite their high digestibility, CSBM diets contain anti-nutritional components such as arabinoxylans, cellulose, galactomannans, and heat-stable protein fractions, which can increase digesta viscosity, reduce nutrient accessibility, and impair protein digestion, thereby limiting overall nutrient utilization [8,9,10,11].

Exogenous enzymes are commonly employed to mitigate these limitations. Xylanase hydrolyzes arabinoxylans, reducing intestinal viscosity and improving nutrient accessibility, metabolizable energy utilization, and growth performance [8,12,13,14,15]. Protease enhances protein hydrolysis, reduces endogenous nitrogen losses, and contributes to skeletal development by enhancing the availability of amino acids required for collagen synthesis, which provides the structural framework of the bone matrix and supports mineralization and mechanical strength [11,16,17].

Combined supplementation of carbohydrases and proteases may provide additive or synergistic benefits by improving carbohydrate and protein digestion simultaneously [18]. In CSBM diets, substantial nutrient fractions (primarily starch, proteins, lipids, and minerals) are physically encapsulated within complex non-starch polysaccharides (NSP)-rich cell walls; xylanase-mediated disruption of these matrices increases accessibility of entrapped protein, enhancing protease efficacy [14,15,18]. This facilitates efficient nutrient release and utilization, which is critical for meeting the metabolic demands of fast-growing broilers.

While many studies have reported performance benefits of enzyme supplementation, few have simultaneously evaluated effects on intestinal viscosity, cecal microbial activity, meat quality attributes, and skeletal characteristics [19,20]. Traditional bone assessments, relying on linear measurements or ash content, may not fully capture functional adaptation under intensive growth conditions. Advances in geometric morphometrics and volumetric bone analyses now allow detailed evaluation of bone shape, architecture, and structural quality [21,22,23,24].

Therefore, this study aimed to investigate the effects of dietary xylanase and protease, individually or combined, on growth performance, intestinal viscosity, intestinal histomorphology, cecal microbial populations, and cecal volatile fatty acid profiles as well as on breast meat quality and skeletal development (*tibiotarsus*, *femur*, and *tarsometatarsus*) in broiler chickens fed CSBM-based diets. We hypothesized that enzyme supplementation would enhance digestive efficiency, nutrient utilization, and gut health, thereby improving growth performance while supporting meat quality and skeletal integrity. By integrating performance, intestinal, microbial, meat quality, and skeletal parameters, this study provides a comprehensive evaluation of the nutritional value of xylanase and protease supplementation in modern broiler production systems.

## 2. Materials and Methods

### 2.1. Animals and Experimental Design

The study was approved by the Animal Care and Use Committee of Ankara University, Türkiye (2025-14-161).

A total of 540 daily Ross 308 (Beypiliç A.Ş., Bolu, Türkiye) male broiler chicks were allocated into six groups, each containing 90 chicks. Each group was further divided into six replicates (subgroups), with 15 chicks per replicate. Each replicate was placed in separate pens (120 × 120 cm). Wood shavings were used as litter in each pen. Feed and water were provided for ad libitum consumption, and the diets were presented in mash form. A 23 h light and 1 h dark cycle was applied in the first week of the trial. After the first week, the dark period was gradually increased to 6 h within a week, and the 18 h of light and 6 h of dark cycle was applied until the 42nd day. Room temperature was 32 ± 2 °C during the first week and gradually reduced to an average of 24–26 °C, which was maintained until slaughter age. The control group (C) received no additives. The diets of the treatment groups were supplemented with the enzymes at the following inclusion levels: 100 g/t xylanase (X100), 200 g/t xylanase (X200), 250 g/t protease (P250), 500 g/t protease (P500), and a combination of 100 g/t xylanase + 250 g/t protease (XP). The xylanase product (AllAmino XTRA; Greenlife Bio Technology Company, Afyonkarahisar, Türkiye) was a fermented, feed-grade enzyme preparation containing endo-1,4-β-xylanase produced by *Cellulosimicrobium cellulans*, with a declared enzymatic activity of 22,000 U/g product. Accordingly, the dietary xylanase activities were 2.2 × 10^6^ U/t (X100) and 4.4 × 10^6^ U/t (X200). The protease product (AllAmino PRO; Greenlife Bio Technology Company, Afyonkarahisar, Türkiye) was a fermented, feed-grade enzyme preparation consisting of a mixture of proteolytic enzymes, including subtilisin A, proteinase K, trypsin, α-chymotrypsin, and protease from *Aspergillus oryzae* produced by *Bacillus subtilis*. The declared total enzymatic activity was 18,500 U/g product, corresponding to dietary protease activities of 4.63 × 10^6^ U/t (P250) and 9.25 × 10^6^ U/t (P500). In the combined treatment (XP), xylanase and protease were supplied at 2.2 × 10^6^ U/t and 4.63 × 10^6^ U/t, respectively.

#### Sample Size Determination

For performance traits, pens were considered the experimental unit (6 pens/treatment; overall: 6). For other studies, two birds were sampled per pen (12 birds/treatment; overall: 72). Sample size adequacy was evaluated by power analysis for one-way ANOVA at α = 0.05. The available sample size provided 80% power to detect large effects for pen-based outcomes (Cohen’s f ≈ 0.65) and moderate-to-large effects for bird-based carcass traits (Cohen’s f ≈ 0.44).

### 2.2. Experimental Diets

Commercial diets formulated according to the Ross 308 catalog [25] were procured from a commercial company, consisting of a starter diet for the chick starter phase (0–14 days), a grower diet for the growing phase (15–28 days), and a finisher diet for the finishing phase (29–42 days). Ingredients and composition of the basal diets are given in Table 1.

### 2.3. Data Collection and Measurements

The nutrient composition of the diets was analyzed according to AOAC methods [26]. For the analysis of calcium and total phosphorus, diet samples were subjected to wet digestion prior to inductively coupled plasma-mass spectrometry (ICP-MS) measurement, following established procedures for animal feed analysis [27]. Approximately 0.2 g of finely ground feed sample was placed into PTFE microwave digestion vessels, and concentrated nitric acid (65%) together with hydrogen peroxide (30%) was added. Samples were allowed to pre-digest at room temperature for 30 min before microwave treatment. Microwave digestion was performed using a CEM MARS 5 system (CEM Corporation, Matthews, NC, USA) with a multi-step program, including a temperature ramp to 180–210 °C over 20 min and a holding time of 20 min at the target temperature. After cooling to room temperature, the digested samples were diluted to 50 mL with ultrapure water. ICP-MS (Agilent 7500ce; Yokogawa Analytical Systems, Tokyo, Japan) was tuned and operated according to the manufacturer’s recommendations and AOAC-based methodologies, with radiofrequency power set at approximately 1500–1600 W, argon as the plasma gas, and helium collision mode applied to minimize spectral interferences [27]. Metabolizable energy was estimated using the equation described by Erol et al. [28].

#### 2.3.1. Performance Parameters

The body weights of the birds were determined by weighing the individually at the beginning of the study (day 0), and at the times of 7, 14, 21, 28, 35, and 42 days. Weight gains were calculated from the differences between the weights. Feed intake (FI) was determined as a subgroup. Feed conversion ratio (FCR) was calculated as kilograms of feed consumed per kilogram of weight gain. Birds were monitored daily for general health and welfare. European production efficiency factor (EPEF) values were calculated according to Onbaşılar et al. [29]. Performance parameters were evaluated on a pen (subgroup) basis.

#### 2.3.2. Carcass, Organ, and Meat Parameters

On the 42nd day of the trial, twelve broilers per treatment (two birds from each pen) were individually weighed and slaughtered by jugular vein incision. Hot carcass weight, liver, heart, gizzard, spleen, bursa Fabricius, and abdominal fat were recorded to calculate the carcass yield and relative weights of internal organs.

The left breast meat samples were appropriately excised and cut into uniform slices. Each breast sample was divided into three equal sections (upper, middle, and lower). The upper portion was used for water-holding capacity (WHC) analysis, the lower portion for antioxidant measurements, and the middle portion for proximate composition analysis according to AOAC [26].

WHC of breast meat was evaluated based on the amount of water released after centrifugation, using a modified procedure adapted from a previously described method [30], similar to the expressible juice method described by Onbaşılar et al. [31]. Samples collected from the same anatomical location were weighed into 15 mL centrifuge tubes containing filter paper and centrifuged at 2500× *g* for 20 min at 4 °C. All analyses were completed within 24 h after sampling. WHC was expressed as the percentage of released water and calculated using the formula: 100 × ((weight before centrifuge − weight after centrifuge)/weight before centrifuge). Higher released water values were interpreted as lower WHC.

##### Antioxidant Characteristics of Breast Meat

Breast meat samples (1 g) were transferred into 15 mL centrifuge tubes, and 5 mL of distilled water was added. The samples were vortex-mixed for 1 min and subsequently homogenized for 30 s using an ultrasonic homogenizer. Afterward, 3.5 mL of chloroform was added, followed by additional vortexing and homogenization. The resulting homogenates were centrifuged at 2100× *g* for 15 min, and the supernatants were collected for the analysis of total phenolic content (TPC) and 2,2 diphenyl-1-picrylhydrazyl (DPPH) radical scavenging activity (RSA) [32].

TPC in the extracts was quantified using the Folin–Ciocalteu assay [33] with gallic acid as the reference standard as reported in Onbaşılar et al. [31]. Absorbance was measured at 760 nm using a spectrophotometer, and results were calculated from a standard curve (y = 5.8895x, R^2^ = 0.9945), where y denotes absorbance and x represents gallic acid concentration. TPC values were expressed as mg gallic acid equivalents (GAE) per g of sample.

DPPH RSA was assessed by measuring absorbance at 517 nm as reported in Onbaşılar et al. [31]. The inhibition % of DPPH was calculated as follows. [1 − (absorbance of sample/absorbance of control)] × 100.

For the evaluation of total antioxidant status (TAS) and total oxidant status (TOS), 1 g of breast meat sample was homogenized with 9 mL of 140 mM KCl solution for 30 s using an ultrasonic homogenizer and vortexed for 1 min. The homogenates were centrifuged at 2100× *g* for 10 min, and the supernatants were collected for analysis. TAS (mmol Trolox equivalent (TE)/kg) and TOS (µmol H_2_O_2_ equivalent/kg) were determined by colorimetric methods using commercial assay kits (Rel Assay Diagnostics, Gaziantep-Türkiye, Cat No: RL0017 and RL0024, respectively). The oxidative stress index (OSI) values were calculated as follows: (TOS, µmol/TAS, µmol) × 100, as described by Onbaşılar et al. [31].

#### 2.3.3. Intestinal Digesta Viscosity

Jejunal and ileal digesta samples were thoroughly homogenized and centrifuged at 5000× *g* for 10 min at 4 °C. An aliquot of 0.5 mL the resulting supernatant was used to determine digesta viscosity using a digital viscometer (DV-II + Pro, Brookfield Engineering Laboratories Inc., Stoughton, MA, USA). Viscosity values were expressed in centipoise (cP) as stated by Yalçın et al. [34].

#### 2.3.4. Intestinal Morphological Characteristics

Segments from the duodenum, jejunum, and ileum were collected for morphological evaluation. Tissue samples were rinsed twice with 0.1 M phosphate buffer and subsequently fixed in 3% glutaraldehyde for 24 h. After fixation, the samples were dehydrated using a graduated acetone series (25, 50, 70, and 100%). From each intestinal segment, two representative areas (inner and longitudinal surfaces) were excised and mounted on specimen stubs. Morphological examinations were performed using a scanning electron microscope (Quanta FEG 250, FEI, Hillsboro, OR, USA), which allows direct imaging without vacuum application, critical drying, or gold coating. Villus height (VH) and crypt depth (CD) measurements were obtained from the images as µm, and the villus height-to-crypt depth ratio (VH/CD) was calculated for each sample [35].

#### 2.3.5. Cecal Volatile Fatty Acid Profile and Microbial Populations

Following slaughter, the paired ceca were carefully removed. One cecal pouch was allocated for volatile fatty acid (VFA) analysis, while the other was used for the determination of microbial populations. Each sample was placed into sterile 50 mL tubes and immediately stored at −18 °C until further analysis.

For VFA determination, cecal samples were thawed at 4 °C and thoroughly homogenized. Approximately 0.5 g of homogenate was used for dry matter analysis. The remaining portion was diluted with distilled water, rehomogenized, and centrifuged at 4000× *g* for 15 min at 4 °C. An aliquot (1 mL) of the supernatant was transferred into Eppendorf tubes, mixed with metaphosphoric acid solution, and allowed to precipitate in an ice bath for 30 min. After centrifugation at 11,000× *g* for 10 min, the clarified supernatants were collected for VFA quantification. VFAs were analyzed using a gas chromatograph (GC-2000, Shimadzu, Kyoto, Japan) equipped with a flame ionization detector (FID) and a TRB-FFAP capillary column (Teknokroma, Barcelona, Spain; 30 m × 0.53 mm internal diameter). Injector and detector temperatures were maintained at 230 °C and 250 °C, respectively. The oven temperature was held at 120 °C for 4 min, increased to 160 °C at a rate of 4 °C/min, and maintained for an additional 4 min. Helium was used as the carrier gas, and the injection volume was 1 µL as described by Yalçın et al. [36]. Results were expressed as mmol per g of cecal digesta dry matter (DM) and as proportions (%) of total VFA (the sum of individual short-chain fatty acids including acetic acid, propionic acid, isobutyric acid, butyric acid, isovaleric acid, valeric acid, isocaproic acid, and caproic acid). The branched-chain fatty acids (BCFA) fraction was defined as the sum of isobutyric acid, isovaleric acid, and isocaproic acid concentrations.

For microbial enumeration, cecal digesta were homogenized, and 1 g of sample was suspended in 9 mL of sterile physiological saline solution. Serial tenfold dilutions were prepared up to 10^−8^, and 100 µL aliquots were spread onto selective agar media in triplicate. Coliform bacteria were enumerated on MacConkey agar (Merck, Darmstadt, Germany) [37], whereas *Lactobacillus* spp. were cultured on de Man, Rogosa, and Sharpe (MRS) agar (Merck, Darmstadt, Germany) [38]. Plates were incubated at 37 °C for 24–48 h. Colonies were counted, averaged across replicates, and results were expressed as log_10_ colony-forming units (CFU) per g of cecal digesta.

#### 2.3.6. Computed Tomography (CT), Three-Dimensional (3D) Modelling and Geometric Morphometric Analysis

##### Three-Dimensional Reconstruction

3D representations of anatomical structures were generated from CT images. Scanning was performed using a 256-slice CT scanner under standardized acquisition conditions, with a slice thickness of 0.6 mm. Image datasets were transferred to the 3D Slicer software environment (version 5.8.0) for subsequent segmentation and reconstruction [39].

A semi-automated segmentation approach was employed to obtain three-dimensional reconstructions of the anatomical components [40]. To ensure consistency across serial sections, a fixed intensity threshold was applied throughout the segmentation process. All datasets were processed using identical software parameters, without additional adjustments. Final 3D models were produced by integrating axial, sagittal, and coronal views. When required, manual refinement and visual verification were performed to confirm anatomical accuracy.

##### Identification of Landmarks

3D geometric morphometric techniques were applied to assess shape differences among experimental groups. Anatomical landmarks were manually placed within the 3D Slicer platform (version 5.8.0; [41]), ensuring correspondence to homologous locations across all specimens (Table 2). To accurately represent curved surfaces and complex anatomical features, sliding semi-landmarks were also included in the analysis.

Landmarks were classified following the criteria proposed by Bookstein [42]. Type I landmarks represented well-defined anatomical junctions with clear biological relevance. Type II landmarks corresponded to geometrically or functionally distinct points, such as maxima of curvature or anatomical extremities [22].

##### Geometric and Linear Morphometric Analysis

Both geometric and linear morphometric techniques were employed to characterize the *femur*, *tibiotarsus*, and *tarsometatarsus*.

For geometric morphometrics, landmark coordinate datasets were imported into 3D Slicer software (version 5.8.0) and subjected to Generalized Procrustes Analysis (GPA) to remove non-shape variation related to position, orientation, and size. The resulting Procrustes-aligned coordinates were subsequently used for shape comparisons. Principal Component Analysis (PCA) was performed to summarize the main patterns of shape variation and to assess group-related clustering. 3D bone models were generated using the ‘Segment Editor’ module of 3D Slicer. Segmentation was conducted using a semi-automatic, threshold-based approach, with threshold values set between 400 and 3071 HU for masking. Window width and level settings ranged from 700 (W) to 300 (L) to optimize bone visualization. The reconstructed models were manually refined to correct segmentation artifacts and ensure anatomical accuracy. Shape changes along principal component axes were visualized directly on 3D models. Lollipop plots were generated to illustrate the direction and magnitude of landmark displacements relative to the mean shape. The statistical procedures underlying PCA and shape deformation visualization are described in the Statistical Analysis section. The visualizations were used for descriptive interpretation of shape variation rather than for formal hypothesis testing [41].

Linear morphometric assessments were conducted in accordance with established osteometric guidelines described by von den Driesch [43]. A total of nine parameters were recorded: GL (total bone length), BP (maximum proximal breadth), BD (maximum distal breadth), CEDG (minimum shaft width), CEGG (maximum shaft width), CCR (cranial cortical thickness), CCD (caudal cortical thickness), CL (lateral cortical thickness), and CM (medial cortical thickness) [43]. All measurements were obtained digitally from high-resolution 3D bone models reconstructed following segmentation procedures. Anatomical reference planes were standardized across samples to ensure consistency and reproducibility of measurements.

##### Bone Measurements

Bone surface area (mm^2^) and volume (cm^3^) were calculated from the reconstructed 3D models using 3D Slicer software [39]. Individual bone weights were determined using a precision digital balance with a sensitivity of 0.01 g.

#### 2.3.7. Statistical Analyses

Prior to statistical evaluation, data distribution was examined for normality using the Shapiro–Wilk test, and variance homogeneity was assessed with Levene’s test. Performance variables, meat quality traits, and linear bone measurements were analyzed using one-way analysis of variance (ANOVA). When significant treatment effects were observed, group means were compared using Tukey. Statistical significance was declared at *p* < 0.05.

The study comprised six experimental groups (Control, X100, X200, P250, P500, and XP). To evaluate dose–response relationships, two numeric dose variables were generated based on group assignment: Xylanase (XYL) dose (0, 100, and 200) and Protease (PRO) dose (0, 250, and 500). Two-way ANOVA is implemented within the general linear model (GLM) framework with XYL dose and PRO dose as fixed factors. Main effects of XYL and PRO and their interaction (XYL × PRO) were examined. The absence of a significant XYL × PRO interaction suggests that the combined effect of XYL and PRO is additive, indicating neither synergistic nor antagonistic interaction.

To enhance the robustness of the statistical inference for linear morphometric variables, permutation tests were additionally applied as a non-parametric alternative, particularly when deviations from parametric assumptions were suspected. All linear morphometric analyses were conducted across nine bone dimensions, and comparable trends were observed between parametric and non-parametric approaches.

Geometric morphometric analyses were performed using the PAST software package (version 4.03) [44]. Shape differences among groups were evaluated using ANOVA based on Procrustes-aligned coordinates. Where appropriate, permutation-based testing was employed to confirm the reliability of the results.

## 3. Results

### 3.1. Performance Parameters

The effects of different enzyme types and inclusion levels on broiler performance parameters across experimental periods are illustrated in Supplementary Figure S1 and summarized in Table 3. The initial BW of the chicks was 45.31 ± 0.04 g, and no statistically significant differences were observed among the groups, confirming the homogeneity of chicks at the beginning of the trial (Table 3). Overall ANOVA indicated a significant treatment effect on BW at 42 days of age (*p* = 0.004). The effect was primarily driven by xylanase supplementation and the xylanase-protease combination, while protease supplementation alone did not significantly differ from the control group. Two-way ANOVA revealed a significant main effect of xylanase (*p* = 0.003), whereas the main effect of protease was not significant (*p* = 0.088). However, a significant xylanase × protease interaction was detected (*p* = 0.048), indicating that the effect of protease on final BW depended on the presence of xylanase.

Body weight gain (BWG) during days 1–21 tended to be higher in enzyme-supplemented groups compared with the control group; however, this difference was only marginally significant (*p* = 0.051). Overall ANOVA revealed a significant treatment effect on BWG during days 22–42 (*p* = 0.008) as well as over the entire experimental period (*p* = 0.004). During the 22–42 day growth period, BWG in the X100 and XP groups was significantly higher than that of the control group, whereas protease-supplemented groups did not differ statistically from the control. Similarly, over the overall experimental period (days 1–42), BWG was significantly enhanced in xylanase-supplemented groups and in the combined xylanase-protease group compared with the control, while protease supplementation alone did not result in a significant improvement in BWG. Consistent with these findings, two-way ANOVA demonstrated a significant main effect of xylanase on BWG during days 22–42 and over the overall experimental period (*p* = 0.003), whereas the main effect of protease remained non-significant. A significant xylanase × protease interaction was detected only for cumulative BWG (*p* = 0.049), indicating that the response to protease supplementation depended on the presence of xylanase over the full production cycle (Table 3).

FI was significantly affected by dietary treatments during all experimental periods (*p* < 0.01). During days 1–21, FI differed among groups (*p* = 0.003), with a significantly higher intake observed only in the combined xylanase-protease group compared with the control, while the other enzyme-supplemented groups did not differ statistically. During days 22–42, FI was significantly increased in the X100 group, whereas a significant decrease was observed in the P250 group compared with the control. Over the entire experimental period (days 1–42), FI followed a pattern comparable to that observed during days 22–42. Two-way ANOVA demonstrated a significant main effect of xylanase on FI during days 22–42 and over the overall experimental period (*p* < 0.001), whereas the main effect of protease was not significant. No significant xylanase × protease interaction was detected for FI across all periods (Table 3).

FCR was significantly influenced by dietary treatments during all experimental periods (*p* < 0.05). During days 1–21, FCR was significantly lower in the X200 group compared with the control (*p* < 0.05). During days 22–42, significantly lower FCR values were observed in the X100, P250, P500, and XP groups (*p* < 0.05). Over the overall experimental period, all enzyme-supplemented groups exhibited significantly lower FCR values compared with the control. Two-way ANOVA indicated a significant main effect of xylanase on FCR during all experimental periods, whereas the main effect of protease was significant during days 22–42 and over the overall experimental period. In addition, a significant xylanase × protease interaction was detected for FCR in each experimental period, although the magnitude and direction of the interaction varied depending on the period (Table 3).

All enzyme-supplemented groups exhibited significantly higher EPEF values compared with the control group (*p* = 0.003), indicating an overall improvement in production efficiency with enzyme inclusion. Two-way ANOVA revealed significant main effects of both xylanase and protease on EPEF (*p* = 0.007 and *p* = 0.012, respectively). In addition, a significant xylanase × protease interaction was observed (*p* = 0.033), indicating that the effects of the two enzymes on production efficiency were not simply additive (Table 3).

### 3.2. Carcass Yield and Relative Organ Weights

The effects of dietary enzyme supplementation at different levels on carcass yield and relative internal organ weights are presented in Table 4. Enzyme inclusion had no significant effects on any of the percentages of carcass or organ parameters evaluated (*p* > 0.05). Carcass yield ranged between 74.71% and 75.39% across all groups, with no meaningful differences among treatments.

### 3.3. Intestinal Viscosity

The effects of different enzyme supplementations at different levels on intestinal viscosity in broilers are presented in Table 5. In the jejunum, broilers fed the control diet exhibited the highest viscosity value (1.46 cP). Supplementation with xylanase at both 100 g/t (X100) and 200 g/t (X200), as well as the combined xylanase + protease treatment (XP), resulted in significantly lower jejunal viscosity compared with the control group (*p* < 0.001). Protease supplementation alone (P250 and P500) numerically reduced jejunal viscosity; however, these reductions did not differ significantly from the control group.

All enzyme-supplemented groups showed significantly lower ileal viscosity values compared with the control diet (*p* < 0.05). No statistically significant dose–response effect was detected for protease supplementation, as increasing the inclusion level from P250 to P500 did not result in further reductions in either jejunal or ileal viscosity. For xylanase, increasing the dose from 100 to 200 g/t did not lead to a significant additional reduction in jejunal viscosity, whereas ileal viscosity values were numerically lower at the higher xylanase dose. Two-way ANOVA revealed significant main effects of xylanase on both jejunal and ileal viscosity (*p* < 0.001 for both), indicating a strong viscosity-reducing action of xylanase. Protease also showed a significant main effect on viscosity in both intestinal segments (*p* = 0.034 for the jejunum and *p* = 0.001 for the ileum). However, the xylanase × protease interaction was not significant for either the jejunum or ileum (*p* > 0.05), suggesting that the reductions in intestinal viscosity were attributable to the independent and likely additive contributions of xylanase and protease rather than a synergistic interaction.

### 3.4. Cecal Volatile Fatty Acid Composition

The effects of different enzyme supplementations on cecal VFA composition in broilers are presented in Table 6. In terms of absolute concentrations, dietary enzyme supplementation significantly increased the concentrations of acetic, propionic, butyric, and isobutyric acids, as well as total VFA and total BCFA concentrations, compared with the control group (*p* < 0.05). In contrast, isovaleric, isocaproic and caproic acid concentrations were not significantly affected by dietary treatments (*p* > 0.05).

When VFAs were expressed as proportions of total VFA, dietary enzyme supplementation significantly altered the relative fermentation products. The proportion of acetic acid was significantly lower in all enzyme-supplemented groups compared with the control (*p* < 0.001), whereas the proportion of butyric acid was significantly increased, particularly in the X100 and XP groups (*p* = 0.013). The relative proportion of valeric acid was significantly higher in the XP group compared with the control (*p* = 0.049). However, the proportions of propionic acid and isovaleric acid, as well as the percentage of total BCFA, were not significantly influenced by dietary treatment (*p* > 0.05). The acetate to propionate (A/P) ratio was significantly reduced by enzyme supplementation (*p* = 0.001), indicating a shift in cecal fermentation patterns.

Two-way ANOVA revealed significant main effects of xylanase on most individual VFAs, total VFA concentration, total BCFA, and the A/P ratio (*p* < 0.05), highlighting the prominent role of xylanase in modulating cecal fermentation. Protease supplementation exerted significant main effects on VFAs concentration and total VFA concentration (*p* < 0.05), but not total BCFA concentration. Significant xylanase × protease interactions were detected for A/P ratio (*p* = 0.036) and the proportion of acetic acid (*p* = 0.032). For other individual VFAs and total VFA concentration, interaction effects were not significant (*p* > 0.05), indicating that enzyme supplementation exerted independent effects on these parameters.

### 3.5. Cecal Microflora

The effects of dietary enzyme supplementation on cecal microbial populations are presented in Table 7. Broilers fed the control diet exhibited the highest coliform count (6.68 log_10_ CFU/g). Supplementation with xylanase at 100 g/t (X100) and 200 g/t (X200), as well as the combined xylanase + protease treatment (XP), resulted in significantly lower coliform counts compared to the control group (*p* < 0.05). In contrast, protease supplementation alone (P250 and P500) resulted in intermediate coliform counts that did not differ significantly from other groups. Two-way ANOVA revealed a significant main effect of xylanase on coliform counts (*p* = 0.002), whereas the main effect of protease and the xylanase × protease interaction were not significant (*p* > 0.05), indicating that reductions in coliform populations were primarily associated with xylanase supplementation.

In contrast, *Lactobacillus* spp. counts were significantly increased by dietary enzyme supplementation (*p* < 0.001). Two-way ANOVA demonstrated significant main effects of both xylanase (*p* = 0.001) and protease (*p* < 0.001) on *Lactobacillus* spp. counts, while the xylanase × protease interaction was not significant (*p* = 0.725). These results indicate that xylanase and protease independently contributed to increased *Lactobacillus* spp. populations, without evidence of a synergistic interaction or a clear dose–response relationship within the tested inclusion levels.

### 3.6. Intestinal Histomorphological Parameters

Significant differences among experimental groups were observed for several intestinal histomorphological parameters (*p* < 0.05), as presented in Table 8.

In the duodenum, the values for villus height and crypt depth were lowest in the control group and were significantly increased in all enzyme-supplemented groups (*p* < 0.05). Two-way ANOVA revealed significant main effects of both xylanase and protease on villus height and crypt depth (*p* < 0.001). A significant xylanase × protease interaction (*p* < 0.001) was detected for villus height, whereas their interaction was not significant for crypt depth. Consequently, the villus height to crypt depth ratio was significantly higher in the X100, X200, P250, and XP groups compared with the control (*p* < 0.001). Two-way ANOVA indicated significant main effects of xylanase (*p* < 0.001) and protease (*p* = 0.002), as well as a significant xylanase × protease interaction (*p* < 0.001) for the VH/CD ratio (*p* < 0.001).

In the jejunum, dietary enzyme supplementation significantly increased villus height compared with the control group (*p* < 0.001), with the highest value observed in the XP group. Two-way ANOVA showed significant main effects of both xylanase and protease on villus height (*p* < 0.001), while the xylanase × protease interaction was not significant (*p* = 0.085). Crypt depth also differed significantly among dietary treatments (*p* < 0.001), with the highest value recorded in the P500 group and the lowest in the X100 group. Two-way ANOVA indicated that both xylanase (*p* = 0.003) and protease (*p* < 0.001) exerted significant main effects on crypt depth, and a significant xylanase × protease interaction was detected (*p* < 0.001). The VH/CD ratio was significantly higher in xylanase-supplemented groups, the P250 group, and the XP group compared with the control (*p* < 0.001). Two-way ANOVA confirmed significant main effects of xylanase (*p* < 0.001) and protease (*p* = 0.004), along with a significant interaction between the two enzymes (*p* < 0.001).

In the ileum, villus height was significantly higher in protease-supplemented groups, the X200 group, and the combined xylanase + protease group compared with the control (*p* < 0.001), with the highest value observed in the XP group. Two-way ANOVA indicated significant main effects of both xylanase and protease on villus height (*p* < 0.001), whereas the xylanase x protease interaction was not significant (*p* = 0.136). Crypt depth was significantly affected by dietary treatments (*p* < 0.001), with the lowest value observed in the X100 group, and the highest in the P500 group. Two-way ANOVA revealed significant main effects of xylanase and protease (*p* < 0.001), as well as a significant xylanase × protease interaction (*p* = 0.001). The VH/CD ratio was significantly improved in the X100 and XP groups compared with the control (*p* < 0.001). Two-way ANOVA indicated a significant main effect of xylanase (*p* = 0.002), whereas the main effect of protease was not significant (*p* = 0.198); however, a significant xylanase × protease interaction was detected (*p* = 0.011).

### 3.7. Breast Meat Properties

The effects of xylanase and protease supplementation at different inclusion levels on WHC, proximate composition, and antioxidant parameters of broiler breast meat were not statistically significant (*p* > 0.05) as shown in Table 9. WHC values ranged from 19.09% to 20.81% among the experimental groups. Crude protein content varied between 22.38% and 22.74%, indicating no treatment effect. TPC ranged from 0.96 and 1.09 mg GAE/g, while DPPH RSA varied between 69.07% and 71.75%. OSI remained statistically unchanged across the groups, with values between 0.121 and 0.147.

### 3.8. Geometric and Linear Bone Morphometric Analysis

Significant differences among experimental groups were observed for multiple linear morphometric parameters of the *femur*, *tibiotarsus* (except total length), and *tarsometatarsus* (*p* < 0.05; Table 10).

For the *femur*, total length was significantly greater in the X100, X200 and P500 groups compared with the control group (*p* < 0.05). Proximal *femur* width was significantly increased only in the X200 group relative to the control (*p* < 0.05). Distal *femur* width was significantly greater in the P250, P500, and XP groups (*p* < 0.05), while X100 and X200 groups did not differ statistically from other groups. The narrowest corpus width was significantly increased in all enzyme-supplemented groups (*p* < 0.05). In contrast, the longest corpus width was significantly higher in the X100 group compared with the control, P500 and XP groups (*p* < 0.05), with no consistent dose-related trend. Cortical thickness parameters exhibited site-specific responses. Cranial and caudal corpus thickness were significantly higher in the P250 group compared with the control (*p* < 0.05), while lateral corpus thickness was highest in the P500 group and medial corpus thickness was highest in the P250 group (*p* < 0.05). Two-way ANOVA revealed significant main effects of both xylanase and protease for most femoral measurements (*p* < 0.05). Significant xylanase × protease interactions were detected for femoral parameters, except proximal width and medial corpus thickness, indicating that combined supplementation influenced specific aspects of femoral morphology in a parameter-dependent manner.

For the *tibiotarsus*, total length was not significantly affected by dietary treatments (*p* > 0.05). Proximal width and narrowest corpus width were significantly increased in the P500 group (*p* < 0.001). Medial corpus thickness was significantly increased in all enzyme-supplemented groups, while xylanase-supplemented groups and the P250 group also exhibited increased lateral and caudal corpus thickness. Two-way ANOVA revealed significant main effects of both xylanase and protease on most *tibiotarsus* width and cortical thickness parameters (*p* < 0.05), along with significant xylanase × protease interactions for several traits.

For the *tarsometatarsus*, X100 and XP diets resulted in significantly greater total length and proximal width compared with the control group (*p* < 0.05). Cortical thickness parameters were also significantly affected, with the XP group consistently exhibiting the highest medial, cranial, caudal, and lateral corpus thickness values. Two-way ANOVA confirmed significant main effects of xylanase on all tarsometatarsal traits (*p* < 0.05).

**Figure 1:**
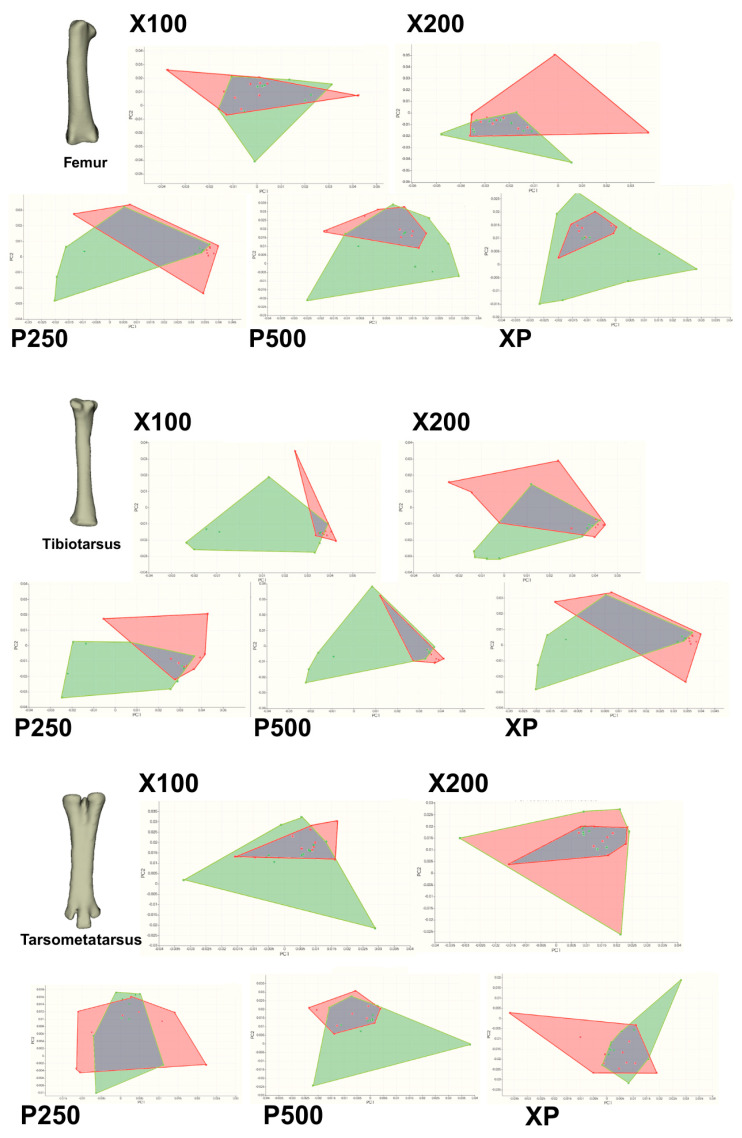
PCA scatter plots for the *femur*, *tibiotarsus* and *tarsometatarsus.* Red dots represent the groups under investigation, while green dots represent the control group. Groups are shown in their own color (red: represent the groups under investigation, green: control groups) within their own boundaries, while intersections are shown in blue. X100: 100 g/t xylanase (2.2 × 10^6^ U/t); X200: 200 g/t xylanase (4.4 × 10^6^ U/t); P250: 250 g/t protease (4.63 × 10^6^ U/t); P500: 500 g/t protease (9.25 × 10^6^ U/t); XP: 100 g/t xylanase (2.2 × 10^6^ U/t) + 250 g/t protease (4.63 × 10^6^ U/t).

**Figure 2:**
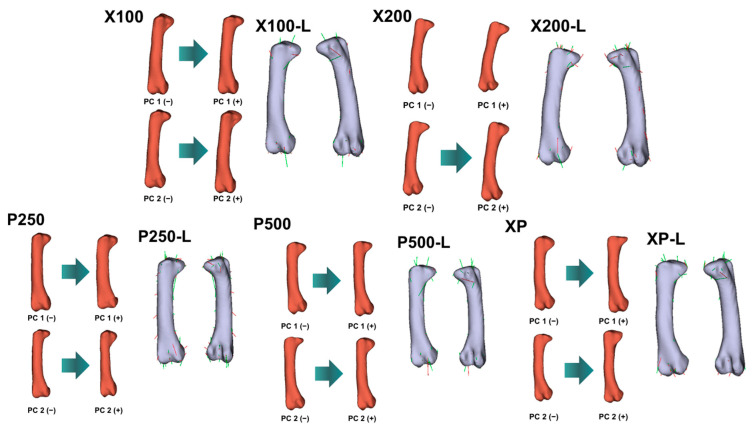
Morphological variations in *femur* shape among experimental groups visualized by principal component (PC) analysis. Red bones represent shape changes along negative (−) and positive (+) extremes of PC1 and PC2, as indicated by arrows. Gray bones (L) represent lollipop graphs illustrating localized shape deformations, where red vectors indicate shape changes associated with PC1 and green vectors indicate shape changes associated with PC2. X100: 100 g/t xylanase (2.2 × 10^6^ U/t); X200: 200 g/t xylanase (4.4 × 10^6^ U/t); P250: 250 g/t protease (4.63 × 10^6^ U/t); P500: 500 g/t protease (9.25 × 10^6^ U/t); XP: 100 g/t xylanase (2.2 × 10^6^ U/t) + 250 g/t protease (4.63 × 10^6^ U/t).

**Figure 3:**
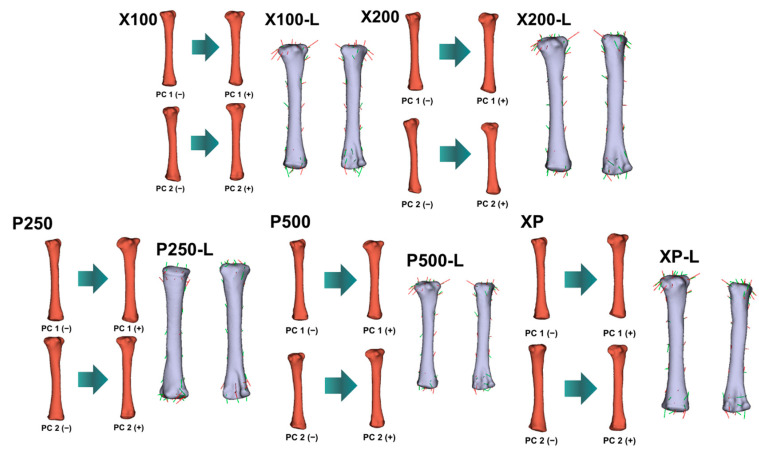
Morphological variations in *tibiotarsus* shape among experimental groups visualized by principal component (PC) analysis. Red bones represent shape changes along negative (−) and positive (+) extremes of PC1 and PC2, as indicated by arrows. Gray bones (L) represent lollipop graphs illustrating localized shape deformations, where red vectors indicate shape changes associated with PC1 and green vectors indicate shape changes associated with PC2. X100: 100 g/t xylanase (2.2 × 10^6^ U/t); X200: 200 g/t xylanase (4.4 × 10^6^ U/t); P250: 250 g/t protease (4.63 × 10^6^ U/t); P500: 500 g/t protease (9.25 × 10^6^ U/t); XP: 100 g/t xylanase (2.2 × 10^6^ U/t) + 250 g/t protease (4.63 × 10^6^ U/t).

**Figure 4:**
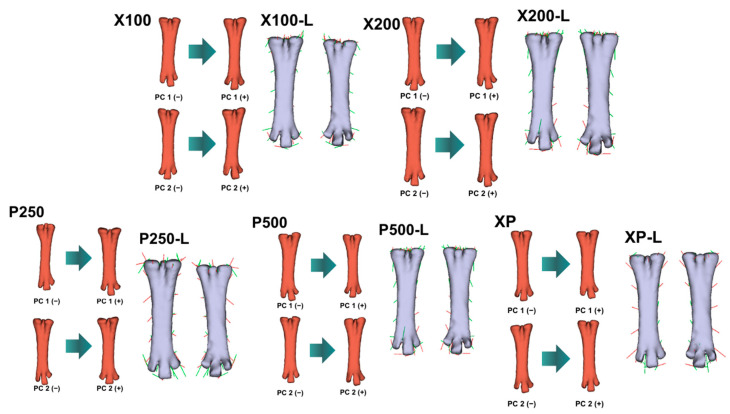
Morphological variations in *tarsometatarsus* shape among experimental groups visualized by principal component (PC) analysis. Red bones represent shape changes along negative (−) and positive (+) extremes of PC1 and PC2, as indicated by arrows. Gray bones (L) represent lollipop graphs illustrating localized shape deformations, where red vectors indicate shape changes associated with PC1 and green vectors indicate shape changes associated with PC2. X100: 100 g/t xylanase (2.2 × 10^6^ U/t); X200: 200 g/t xylanase (4.4 × 10^6^ U/t); P250: 250 g/t protease (4.63 × 10^6^ U/t); P500: 500 g/t protease (9.25 × 10^6^ U/t); XP: 100 g/t xylanase (2.2 × 10^6^ U/t) + 250 g/t protease (4.63 × 10^6^ U/t).

PCA revealed clear shape variation among treatment groups for all evaluated bones (Figure 1, Figure 2, Figure 3 and Figure 4; Table 11). The first two principal components (PC1 and PC2) explained a substantial proportion of the total shape variance, accounting for 37.0–45.4% in the *femur*, 41.9–54.2% in the *tibiotarsus*, and 29.9–37.6% in the *tarsometatarsus*. Notably, for the *tibiotarsus*, the combined contribution of PC1 and PC2 approached or exceeded 50% in several treatment comparisons, suggesting that this bone exhibited a greater sensitivity of shape variation in response to dietary enzyme supplementation compared with the *femur* and *tarsometatarsus*.

The PCA scatter plots (Figure 1) demonstrated clear morphological distinction between enzyme-supplemented and control groups, particularly for the *femur* and *tibiotarsus.* Enzyme-treated groups occupied a broader morphological area, indicating increased shape variability associated with dietary enzyme supplementation. Separation along PC1 was most pronounced in the XP and X200 groups for the *femur*, whereas the P500 and XP groups showed the greatest differentiation in the *tibiotarsus*. In contrast, greater overlap among groups was observed for the *tarsometatarsus*, although enzyme-supplemented birds still exhibited a wider morphological distribution compared with the control group.

Shape deformation (lollipop) graphs (Figure 2, Figure 3 and Figure 4) complemented the PCA results by illustrating the anatomical regions contributing to overall shape variation. For the *femur* and *tibiotarsus*, PC1 primarily longitudinal elongation accompanied by curvature changes along the diaphyseal axis, whereas PC2 reflected more localized variations in bone width and torsional geometry. In contrast, shape variation in the *tarsometatarsus* was less pronounced and mainly involved subtle modifications around the metatarsal condyles, indicating a more conservative morphological response in this bone.

The effects of dietary xylanase and protease supplementation on bone weight, surface area, and volume are presented in Table 12. Enzyme supplementation significantly increased the weight of all evaluated bones (*p* < 0.001). Two-way ANOVA revealed significant main effects of both xylanase and protease on bone weight (*p* < 0.001), with a significant xylanase × protease interaction for femur and *tarsometatarsus* weights (*p* < 0.01), indicating combined effects of enzyme supplementation.

The highest bone weights were consistently observed in the combined enzyme (XP) and high-dose protease (P500) groups, whereas the control group exhibited the lowest values across all bones. Increasing xylanase and protease inclusion levels generally resulted in higher bone weights; however, the magnitude of increase varied among bones, indicating the absence of a clear linear dose–response relationship.

In contrast to bone weight, bone surface area and volume were largely unaffected by dietary treatments for the *femur* and *tarsometatarsus* (*p* > 0.05). For the *tibiotarsus*, enzyme supplementation significantly reduced surface area compared with the control group (*p* = 0.006), with significant main effects of xylanase (*p* = 0.041) and protease (*p* = 0.030), as well as a significant interaction effect (*p* = 0.014). Bone volume of the *tibiotarsus* remained unchanged (*p* > 0.05), suggesting that enzyme-induced modifications primarily influenced bone geometry rather than overall bone volume.

Pairwise comparisons between each enzyme-supplemented group and the control were further evaluated using ANOVA and permutation-based testing (Supplementary Table S1). In the *femur,* highly significant differences were detected for the C-P250 and C-XP comparisons (*p* < 0.001), both associated with high F-values (26.4 and 23.83, respectively), indicating pronounced treatment-induced shape modifications. The C-X200 comparison also showed a significant difference (*p* < 0.05), whereas no significant differences were observed for the C-P500 and C-X100 comparisons (*p* > 0.05). Permutation analyses (*n* = 99.999) confirmed these findings.

In the *tibiotarsus*, significant shape differences were observed for the C-P500, C-X100, C-X200, and C-XP comparisons (*p* < 0.001), whereas the C-P250 group did not differ from the control (*p* > 0.05). For the *tarsometatarsus*, significant differences were limited to the C-X100 and C-XP comparisons (*p* < 0.001), indicating a more treatment-specific morphological response in this bone. These results were consistently supported by permutation-based analyses.

## 4. Discussion

The present study demonstrated that dietary supplementation with xylanase, protease, or their combination influenced broiler growth performance in a phase-dependent manner. Although BWG during the period of 1–21 days was not significantly affected by enzyme supplementation, significant improvements were observed during the 22–42-day growth period, particularly in xylanase-supplemented groups, resulting in higher final BW and cumulative BWG over the entire experimental period. This delayed response suggests that the effectiveness of dietary enzymes becomes more pronounced as birds mature. The lack of significant effects on BWG during the early production phase may be related to the limited FI and immature digestive capacity of young birds, which may constrain the interaction between exogenous enzymes and dietary substrates. As birds age, the naturally increasing FI and enhanced digestive and absorptive capacity likely allow dietary enzymes to exert stronger effects by improving substrate accessibility, fermentation activity, and overall nutrient utilization. Similar age-dependent responses to enzyme supplementation have been reported previously, with variability in the onset and magnitude of growth responses depending on diet composition, enzyme characteristics, and experimental conditions [19,45].

Xylanase supplementation played a dominant role in improving final BW and BWG during the 22–42-day period and across the entire experimental duration, as evidenced by the significant main effect of xylanase in the two-way ANOVA. In contrast, protease supplementation alone did not produce a consistent independent improvement in growth performance. However, the presence of a significant xylanase × protease interactions for final BW and cumulative BWG indicates that the growth response to protease supplementation was dependent on concurrent xylanase inclusion. This interaction suggests that protease efficacy may be enhanced when xylanase-mediated degradation of NSPs improves nutrient accessibility and intestinal conditions, rather than protease acting independently. environment, rather than acting entirely independently.

The improvement in BWG observed during the later production phases is likely associated with enhanced nutrient utilization following enzyme supplementation. Xylanase degrades arabinoxylans in CSBM-based diets, reducing digesta viscosity and improving nutrient diffusion and endogenous enzyme access to substrates [46,47]. Protease supplementation can further enhance protein hydrolysis and amino acid availability while reducing endogenous nitrogen losses [48,49]. Nevertheless, the lack of a consistent additive or pronounced synergistic response in the combined enzyme treatment suggests that the contribution of protease is likely context-dependent and may be limited when xylanase has already enhanced nutrient release in highly digestible CSBM diets. Under such conditions, the potential for additional growth benefits from protease supplementation may be limited, which could help explain the lack of a pronounced synergistic response.

FI responses to enzyme supplementation were also phase-dependent. During days 1–21, FI differed among treatments, with protease supplementation exerting a significant main effect, whereas xylanase did not significantly influence FI. In contrast, during the later phase and across the entire experimental period, both xylanase and protease showed significant main effects on FI, without a significant xylanase x protease interaction. The increased FI observed in xylanase-supplemented groups during the later period may be related to improved gut conditions and reduced digesta viscosity, facilitating feed passage and nutrient utilization efficiency [50]. Conversely, protease supplementation was generally associated with lower or comparable FI relative to the control, suggesting that performance improvements were primarily associated with enhanced nutrient utilization rather than increased FI. This observation is consistent with Freitas et al. [51], who reported unchanged FI despite improved nutrient utilization in protease-supplemented broilers.

Improvements in FCR further support the beneficial effects of dietary enzyme supplementation on feed efficiency. Although enzyme inclusion resulted in more consistent and pronounced improvements during the period of 22–42 days and overall experimental periods, its impact during the first 21 days was less consistent. During the first 21 days, a significant reduction in FCR was observed only in the 200 g/t xylanase group, whereas the remaining enzyme-supplemented treatments showed intermediate responses. These findings suggest that the efficacy of exogenous enzymes on feed utilization becomes more evident as birds age, likely due to increased substrate availability and the progressive maturation of the digestive system. Moreover, the significant main effects and interaction observed for cumulative FCR indicate that feed efficiency was influenced by both individual enzyme supplementation and their combined inclusion, particularly during periods characterized by elevated nutrient demands.

Cowieson and Ravindran [52] have consistently demonstrated that xylanase and protease supplementation can improve FCR in broilers fed CSBM-based diets by enhancing nutrient digestibility and reducing energy losses associated with anti-nutritional factors. Protease supplementation has been reported to improve FCR by 2.32% in broilers [16] while Stefanello et al. [53] observed improvements of 3.4% in BWG and 2.5% in FCR. Vieira et al. [54] also reported reductions in FCR regardless of protease dosage, and Cowieson et al. [48] demonstrated improved BWG and reduced FCR with protease supplementation, even in diets already containing phytase and xylanase. Similarly, Freitas et al. [51] observed enhanced feed efficiency independent of dietary protein and energy levels. In contrast, Lee et al. [11] and Walk et al. [20] reported limited or inconsistent effects of protease supplementation in nutrient adequate diets, highlighting that dietary context strongly influences enzyme efficacy. Exogenous protease supplementation may reduce the antinutritional effects of indigestible protein fractions, thereby improving amino acid utilization and growth performance under appropriate conditions [11,55].

EPEF values were significantly improved by dietary enzyme supplementation, reflecting the combined enhancements in growth performance and feed efficiency. All-enzyme-supplemented groups exhibited higher EPEF values compared with the control, with the greatest improvement observed in the group receiving combined xylanase and protease supplementation. This pattern suggests that the concurrent use of carbohydrase and protease may exert a more pronounced effect on overall production efficiency than the use of either enzyme alone. The superior EPEF values obtained with the combined treatment are indicative of complementary effects between carbohydrase and protease, particularly when performance outcomes are evaluated in an integrated manner rather than through individual parameters [19].

In contrast to the pronounced improvements observed in growth performance and feed efficiency, dietary enzyme supplementation did not significantly affect carcass yield or the relative weights of internal organs in broilers. Carcass yield and the proportions of metabolically active and immune-related organs, including the liver, heart, spleen, bursa of Fabricius, gizzard, and proventriculus, remained comparable among all dietary treatments. These findings indicate that the performance enhancements achieved with enzyme inclusion were not accompanied by changes in carcass yield or disproportionate organ development. Similar results have been reported in previously, where improvements in growth performance following enzyme supplementation were not necessarily reflected in alterations in carcass yield [56] or relative organ weights [57]. Likewise, Lee et al. [11] reported no significant effects of protease supplementation on carcass yield or internal organ proportions in broilers. Abdominal fat percentage was also unaffected by dietary treatments, further indicating that enzyme-related performance improvements were not accompanied by changes in carcass yield or fat accretion [56,57].

The reduction in intestinal viscosity observed with enzyme supplementation represents a key physiological mechanism underlying the improvements in nutrient utilization and growth performance. Elevated digesta viscosity is widely recognized as an important anti-nutritional factor, as it limits enzyme diffusion, slows digesta passage rate, and impairs nutrient absorption [46,47]. In the present study, reductions in jejunal and ileal viscosity were primarily associated with xylanase supplementation. Xylanase-containing diets markedly lowered intestinal viscosity via the hydrolysis of soluble arabinoxylans, thereby reducing WHC and improving the physicochemical environment of the intestine [46,58,59]. In contrast, although protease supplementation exerted a statistically significant main effect on jejunal and ileal viscosity, its impact was less pronounced and more variable than that observed with xylanase, and it was not associated with a clear dose-dependent response. This indicates that the contribution of protease to viscosity modulation is likely indirect rather than driven by direct degradation of NSPs. Protease may instead influence digesta characteristics through improved protein digestion without directly targeting NSP-driven viscosity. In line with this interpretation, Cowieson et al. [48] reported limited direct effects of protease on ileal viscosity of broilers fed corn-wheat-based diets, emphasizing that viscosity-related responses are largely driven by carbohydrase activity. Accordingly, the viscosity-lowering response observed in the present study appears to be mainly attributable to xylanase, with protease contributing to a secondary and context-dependent manner.

Increasing xylanase from 100 to 200 g/t or protease from 250 to 500 g/t did not result in proportional reductions in intestinal viscosity, indicating the absence of a clear-dose-dependent response within the tested inclusion range. This pattern suggests that the viscosity-lowering effect may have approached a plateau, potentially due to limited substrate availability or enzyme-substrate saturation in the gut environment. This apparent plateau may partly explain the absence of a pronounced synergistic effect of combined xylanase and protease supplementation on intestinal viscosity.

Dietary enzyme supplementation resulted in clear improvements in intestinal histomorphology, particularly VH and the VH/CD ratio, with the most pronounced responses observed in the duodenum. These parameters are widely accepted indicators of enhanced intestinal health, epithelial maturation, and absorptive capacity [8,60]. Consistent with previous reports, improvements in VH and VH/CD ratio following protease supplementation have been documented [11,61,62], supporting the role of proteolytic enzymes in promoting mucosal development. In the present study, enzyme supplementation increased duodenal VH, whereas improvements in the VH/CD ratio were primarily driven by xylanase and xylanase + protease supplementation, with protease exerting more variable effects. This response is biologically plausible, as the duodenum represents the primary site for enzymatic digestion and initial nutrient absorption. These morphological adaptations are in agreement with the improved growth performance and feed efficiency observed during the period of 22–42 days and overall periods.

Xylanase-mediated degradation of NSPs likely alleviated digesta viscosity and reduced mechanical and chemical stress on the mucosa, thereby facilitating enterocyte proliferation and villus elongation [47,56]. Similar improvements in duodenal morphology following xylanase supplementation have been previously reported by Ceylan et al. [56]. Both enzymes and combined enzyme contributed to increased villus height and improved VH/CD ratio (except P500) in this segment. While VH was increase in enzyme × supplemented groups, significant increases in VH/CD were observed in X100 and XP groups.

Protease supplementation likely contributed to favorable intestinal morphology by improving protein and amino acid availability, thereby supporting mucosal growth and epithelial renewal [16,48]. In addition, reduced delivery of undigested protein to the hindgut may decrease mucosal irritation and lower maintenance energy demands associated with excessive crypt cell proliferation [48,63].

Dietary enzyme supplementation markedly modulated cecal fermentation characteristics, as evidenced by increased concentrations of total VFAs and major straight-chain VFAs, particularly acetate, propionate, and butyrate. These responses are most plausibly attributed to the increased availability of fermentable substrates arising from NSP hydrolysis in the small intestine, which subsequently enhanced microbial fermentation in the cecum [46,47]. Xylanase exerted the most pronounced effect on total and individual VFAs, whereas protease contributed in a supportive, context-dependent manner. Similar increases in cecal total and individual VFAs following xylanase supplementation have been reported by Singh et al. [64], regardless of dietary wheat bran inclusion, emphasizing the role of xylanase-mediated NSP degradation in promoting microbial fermentation.

The elevation of butyrate concentration observed in xylanase-containing diets is of particular physiological relevance, as butyrate serves as a primary energy source for colonocytes and plays a critical role in maintaining epithelial integrity, barrier function, and immune modulation [65]. Consistent with the present findings, previous studies have reported increases in cecal butyrate and total VFA concentrations following xylanase supplementation [56,66]. Choct et al. [47] also demonstrated that enzyme-induced increases in cecal VFAs primarily reflect enhanced microbial fermentation rather than a direct energetic contribution to the host. Nevertheless, the specific responses of individual VFAs appear to be strongly influenced by dietary composition and fiber characteristics. For instance, increases in acetate, butyrate, and total VFA have been reported in broilers fed corn- or wheat-based diets supplemented with xylanase [67], whereas other studies observed increases mainly in acetate without corresponding changes in propionate or butyrate [68], or increases in butyrate without changes in acetate concentration [69].

Protease supplementation was associated with relatively modest alterations in cecal fermentation, which is consistent with its indirect mode of action. Sanchez et al. [18], reported reduced isobutyric acid concentrations following protease supplementation, likely reflecting decreased nitrogen flow to the hindgut as a result of improved amino acid digestion and absorption in the small intestine. In the present study, total BCFA concentrations were increased by enzyme supplementation, indicating enhanced microbial fermentative activity in the cecum. However, despite this increase, straight chain VFAs remained the dominant fermentation products, suggesting that enzyme supplementation did not induce a disproportionate shift toward excessive proteolytic fermentation and that the overall fermentation profile remained predominantly saccharolytic. Variations in BCFA responses among studies may be attributed to differences in diet composition, enzyme type and dose, and baseline protein digestibility, all of which influence the balance between saccharolytic and proteolytic fermentation in the hindgut.

Enzyme supplementation also influenced the relative fermentation profile, as indicated by a reduction in the acetate-to-propionate (A/P) ratio. A higher relative proportion of propionate is considered metabolically advantageous because propionate is efficiently absorbed and utilized as a gluconeogenic substrate in the liver, supporting systemic energy supply with lower associated energy losses compared with acetate-driven metabolic pathways [70,71,72]. The magnitude of VFA responses was not strictly dose-dependent, suggesting that the fermentation response may have approached a plateau once sufficient fermentable substrate became available to the cecal microbiota. Combined xylanase and protease supplementation generally resulted in numerically higher total VFA concentrations, and a fermentation profile characterized by increased straight-chain VFAs compared with single-enzyme treatments. However, the absence of consistent interaction effects indicates that these responses were predominantly additive rather than synergistic, likely due to overlapping enzymatic mechanisms and constraints related to substrate availability and microbial adaptation. Collectively, the modulation of cecal fermentation observed in this study aligns with enhanced nutrient utilization and underscores the central role of xylanase-driven NSP degradation in shaping hindgut microbial activity in broilers.

Enzyme supplementation favorably modulated the cecal microbial ecosystem, as evidenced by increased *Lactobacillus* spp. populations and reduced coliform bacteria counts. These responses are consistent with previous reports showing that enzyme-induced changes in substrate availability and fermentation dynamics promote beneficial microbial populations while suppressing opportunistic bacteria [47,58,59,73,74]. In the present study, all enzyme-supplemented groups exhibited higher *Lactobacillus* counts than the control, with the highest value observed in the combined xylanase and protease group, reflecting additive contributions of both enzymes rather than a true synergistic interaction.

The reduction in coliform counts, particularly in xylanase-supplemented diets, supports the concept that xylanase-mediated NSP degradation alters nutrient flow to the hindgut and enhances fermentative activity, thereby creating a less favorable conditions for coliform proliferation. Such effects are commonly attributed to competitive exclusion by beneficial bacteria and fermentation-associated reductions in luminal pH driven by increased VFA production [59,66]. In contrast, protease supplementation alone resulted in intermediate coliform counts without significant differences from the control, suggesting that its role in modulating coliform populations was limited and likely indirect under the conditions of the present study.

The observed increase in *Lactobacillus* spp. populations likely reflects complementary mechanisms of action. Xylanase promotes saccharolytic fermentation by increasing the availability of fermentable carbohydrates, whereas protease may influence microbial composition by improving protein digestion and modifying nitrogen flow to the hindgut. Similar increases in cecal *Lactobacillus* populations following xylanase supplementation have been reported previously [56,66], while Van Hoeck et al. [66] additionally reported a reduction in *Escherichia coli* populations. However, the absence of consistent effects on E. coli in the study of Ceylan et al. [56] highlights that microbial responses may vary depending on diet composition, enzyme characteristics, and experimental conditions.

Further support is provided by Singh et al. [64], who demonstrated that xylanase supplementation selectively altered cecal microbial composition without affecting overall microbial diversity, highlighting shifts in community structure rather than species richness. Overall, these findings indicate that dietary enzyme supplementation, particularly xylanase, contributes to a more favorable cecal microbial profile by increasing *Lactobacillus* spp. populations and reducing coliform bacteria, while protease appears to play a supportive role mainly in modulating coliform counts in a context-dependent manner.

Notably, *Lactobacillus salivarius* was significantly enriched in xylanase-supplemented broilers, highlighting the capacity of xylanase to selectively promote beneficial bacterial taxa in the cecum. This finding is consistent with the observed increase in total *Lactobacillus* spp. counts, and the concomitant reduction in coliform populations in the present study. The previous study has similarly demonstrated that xylanase supplementation increases *Lactobacillus* spp. abundance in the broiler cecum, potentially exerting complementary effects by favoring beneficial bacterial communities that suppress pathogenic strains and indirectly support growth performance [59].

Broilers are unable to completely digest and absorb all dietary protein and amino acids, resulting in the passage of undigested protein to the cecum [73,74]. Elevated availability of undigested protein in the hindgut has been associated with increased proliferation of pathogenic or opportunistic bacteria [73,74]. In this context, the enhancement of *Lactobacillus* populations observed in enzyme-supplemented groups may contribute to improved microbial balance by competitively limiting the expansion of potentially harmful bacteria and by modulating the cecal environment toward more favorable conditions.

In the present study, dietary supplementation with xylanase and/or protease did not significantly influence WHC and the proximate composition of broiler breast meat. These findings indicate that the beneficial effects of enzyme supplementation observed at the performance level were primarily associated with improved digestive efficiency and nutrient utilization rather than direct alterations in muscle composition or oxidative balance under the conditions of the present study. The lack of response in breast meat protein content following xylanase supplementation is consistent with the findings of Selim et al. [75], who also reported no significant changes in breast meat protein concentration in broilers fed xylanase-supplemented diets. Similar outcomes were reported by Lee et al. [11], who found that protease supplementation did not affect meat quality in broilers. Kim et al. [76] also observed that xylanase inclusion did not affect WHC, supporting the notion that enzyme-mediated improvements in nutrient digestibility do not necessarily translate into measurable changes in physicochemical meat quality traits. In contrast to the present findings, Skrivan et al. [77] reported an increase in breast meat fat content following xylanase supplementation. Such discrepancies may be attributed to differences in diet formulation, energy density, enzyme characteristics, or broiler genotype, all of which can influence lipid deposition in muscle tissue. Under the conditions of the present study, however, breast meat fat content remained unaffected, indicating that enzyme supplementation did not alter lipid accretion in breast muscle.

Regarding oxidative status, no significant effects of xylanase and/or protease supplementation were detected on TPC, DPPH RSA, TAS, TOS, or OSI in breast meat. These results suggest that enzyme supplementation did not exert a detectable antioxidant effect at the muscle tissue level. Nevertheless, previous studies have demonstrated that xylanase supplementation can enhance antioxidant capacity in other tissues or species. Petry et al. [78] reported improved antioxidant capacity and reduced oxidative stress in pigs receiving xylanase, while Pirgozliev et al. [79] observed enhanced hepatic antioxidant status in broilers. These findings suggest that the antioxidant-related effects of xylanase may be tissue-specific and more pronounced in metabolically active organs such as the liver. The absence of changes in breast meat antioxidant parameters in the present study, therefore, does not preclude systemic antioxidant benefits but rather indicates limited transfer of these effects to muscle tissue.

Dietary supplementation with xylanase and protease, either alone or in combination, exerted bone-specific effects on skeletal morphometry and structure in broilers. The observed responses encompassed not only linear dimensions such as length and width but also cortical thickness, bone weight, and shape characteristics, indicating improvements in skeletal robustness and structural organization rather than simple proportional growth.

Increases in total bone length, particularly in the *femur* and *tarsometatarsus* of xylanase- and XP-supplemented broilers, suggest that enhanced nutrient availability supports longitudinal bone development during rapid growth phases. Xylanase-mediated degradation of NSP reduces digesta viscosity and improves the diffusion and absorption of minerals, especially calcium and phosphorus, which are critical for endochondral ossification, mineral deposition, and longitudinal bone growth [80,81]. Additionally, improved protein digestibility enhances the availability of amino acids required for collagen synthesis, which forms the structural framework of the bone matrix and supports mechanical strength and proper bone morphology. In contrast, the absence of a consistent length response in the *tibiotarsus* indicates that enzymatic effects on longitudinal growth are bone-specific and may depend on growth timing, functional loading, and local mineral turnover rates.

Beyond bone length, marked increases in proximal, distal, and corpus widths—especially in protease-supplemented groups—indicate lateral bone expansion rather than elongation. Such changes are typically associated with enhanced periosteal apposition, a key determinant of bone strength and resistance to mechanical stress [82]. This interpretation is supported by the substantial increases observed in cortical thickness parameters of the *tibiotarsus* and *femur*. Site-specific increases in cranial, caudal, lateral, and medial cortical thickness suggest enhanced osteoid formation followed by more effective mineral deposition, particularly in weight-bearing bones.

Protease supplementation likely contributed to these responses by increasing amino acid availability, thereby supporting collagen matrix synthesis, which provides the structural framework for subsequent mineralization [49]. This mechanism is especially relevant for the *tibiotarsus,* where mechanical demands favor cortical reinforcement over longitudinal extension. Although *tibiotarsus* bone strength and ash percentage were not directly measured in the present study, previous studies have shown that protease supplementation increased *tibiotarsus* bone strength and ash percentage by 9.72% and 2.65%, respectively [16], and increased *tibiotarsus* weight in broilers [83]. The observed improvements in *tibiotarsus* morphometric and structural traits in our study are consistent with these findings, supporting the role of protease in enhancing structural bone quality.

Geometric morphometric analyses and PCA analyses provided additional insight beyond traditional linear measurements by revealing enzyme-induced shape remodeling. The broader morphospace occupation observed in enzyme-supplemented groups reflects increased skeletal plasticity and adaptive morphological variation. Separation along the primary shape axes in the *femur* and *tibiotarsus* captured coordinated changes in longitudinal geometry and diaphyseal curvature, whereas secondary axes represented localized alterations in width and torsional configuration. These shape modifications indicate a redistribution of bone tissue rather than uniform enlargement.

Notably, broilers receiving high-dose protease and combined enzyme supplementation exhibited the most pronounced divergence from the control group in both shape and mass. This pattern aligns with the marked increases in cortical thickness and bone weight, suggesting that enzyme supplementation influenced not only bone size but also the spatial organization of bone tissue. Such remodelling is generally considered advantageous for biomechanical performance and resistance to skeletal disorders in fast-growing broilers [84].

Differences among the *femur*, *tibiotarsus*, and *tarsometatarsus* further emphasize the bone-specific nature of enzymatic responses. While the *femur* and *tarsometatarsus* were more responsive in terms of length and overall shape variability, the *tibiotarsus* showed greater sensitivity in cortical thickness and mass. These differences likely reflect variations in growth dynamics, mineral deposition patterns, and mechanical loading among individual bones [5].

The positive effects of xylanase on bone characteristics observed in the present study are supported by previous research demonstrating improved bone strength following xylanase supplementation. Skrivan et al. [77] reported increased tibial breaking strength in broilers fed xylanase, indicating enhanced mechanical integrity of the bone. Similarly, improved bone health associated with xylanase supplementation has been attributed to increased mineral retention and bioavailability. Muszynski et al. [85] demonstrated that xylanase supplementation influenced the carbonate-to-phosphate ratio and crystallinity index in bone tissue, reflecting improvements in mineral quality and structural organization in laying hens. Muszynski et al. [85] further reported increased calcium content in the *tibiotarsus*, along with elevated levels of trace elements such as magnesium, manganese, and chromium, all of which play crucial roles in bone metabolism and strength. The beneficial skeletal effects of xylanase are therefore likely mediated through enhanced mineral absorption and improved calcium homeostasis, ultimately leading to superior bone quality [77,85]. When combined with protease, these effects appear to be balanced by improved protein utilization, supporting both the organic matrix and mineral phases of bone.

The superior bone quality parameters observed with protease supplementation may be attributed to its extra-proteolytic effects, as documented by Cowieson and Roos [86] and da Nobrega et al. [16]. These effects may include improvements in calcium and phosphorus digestibility, as previously demonstrated by Farrokhi et al. [87] and Olukosi et al. [88]. In addition, dietary proteases may enhance the utilization of non-protein nutrients by preventing the formation of, or by breaking down, protein-phytate complexes [89]. By targeting the protein component of these complexes, protease supplementation may facilitate phytase activity in the diet, thereby enhancing phytate hydrolysis and reducing its antinutritional effects. Consequently, the bioavailability of phosphorus and calcium, two minerals that are critical for bone formation and mineralization, may be increased, contributing to improved bone structural characteristics [16].

## 5. Conclusions

The present study demonstrates that dietary supplementation with xylanase and protease, applied individually or in combination, supports growth performance and intestinal functionality in broiler chickens fed CSBM-based diets, with particularly pronounced effects observed between 22 and 42 days of age. Improvements in feed efficiency were associated with reduced intestinal digesta viscosity, favorable alterations in intestinal histomorphology, and modulation of cecal microbial populations and volatile fatty acid profiles, indicating improved digestive efficiency and gut health. Carcass yield was not affected by enzyme supplementation. Breast meat physicochemical composition and antioxidant status remained unchanged following dietary enzyme inclusion. In contrast, xylanase and protease supplementation positively influenced skeletal development and bone structural characteristics, as reflected by improvements in morphometric traits of the *tibiotarsus*, *femur*, and *tarsometatarsus*. Overall, these findings suggest that dietary xylanase and protease, applied individually or in combination, represent an effective nutritional strategy for supporting growth performance, intestinal functionality, gut health, and skeletal integrity in modern broiler production systems.

## Figures and Tables

**Table 1:** Ingredients and chemical composition of the basal diets (as fed-basis).

Ingredients (kg/t)	Starter (0–14 d)	Grower (15–28 d)	Finisher (29–42 d)
Maize	525.04	560.05	572.27
Soybean meal, 46% CP	328.87	277.67	245.30
Full-fat soya, 34% CP	100.00	110.00	120.00
Soy oil	14.67	23.59	36.00
Limestone	9.98	9.28	7.68
DCP, 18%	7.50	6.08	6.40
Lysine	3.00	2.84	2.81
Methionine	2.63	2.45	2.28
Salt	2.57	2.60	2.60
Sodium bicarbonate	1.40	1.39	1.43
Vitamin premix ^1^	1.00	1.00	1.00
Mineral premix ^2^	1.00	1.00	1.00
L-threonine	0.94	0.70	0.37
Choline chloride, 60%	0.80	0.75	0.75
Phytase ^3^	0.10	0.10	0.10
Anticoccidial ^4^	0.50	0.50	
**Analyzed composition**			
DM, %	89.30	89.45	89.82
CP, %	22.99	21.06	20.14
ME ^5^, kcal/kg	3080	3151	3238
Calcium, %	0.97	0.94	0.87
Total phosphorus, %	0.79	0.74	0.72
**Calculated composition**			
Digestible methionine	0.550	0.514	0.486
Digestible methionine + cystine	0.882	0.824	0.785
Digestible lysine	1.320	1.210	1.150
Digestible arginine	1.462	1.341	1.271
Digestible threonine	0.908	0.824	0.758
Digestible leucine	1.764	1.652	1.584
Digestible isoleucine	0.958	0.888	0.849
Digestible valine	0.989	0.920	0.880
Digestible tryptophan	0.258	0.237	0.225

^1^ vitamin premix (per 1 kg): contains 11,000,000 IU of vitamin A, 3,500,000 IU of vitamin D_3_, 100 g of vitamin E, 3 g of vitamin K_3_, 3 g of vitamin B_1_, 6 g of vitamin B_2_, 15 g of calcium-D-pantothenate, 1 g of vitamin B_6_, 35 g of niacin, 1.5 g of folic acid, 200 mg of biotin, and 20 mg of vitamin B_12_; ^2^ mineral premix (per 1 kg): contains 30 g of copper, 120 g of manganese, 110 g of zinc, 2 g of iodine, 300 mg of selenium, and 50 g of iron; ^3^ 6-phytase, 10,000 FTU/g; ^4^ ionophor anticoccidial, 120 mg salinomycin sodium/kg; ^5^ calculated; CP: crude protein; DCP: dicalcium phosphate; DM: dry matter; ME: metabolizable energy.

**Table 2:** Description of the landmarks related to the anatomical region list.

Landmark Numbers	Type of Landmark	Anatomical Region	Landmark Numbers	Type of Landmark	Anatomical Region
***Femur***			***Tarsometatarsus***		
1	I	*Fovea ligamentum capitis*	1–15	I	*Cotyla lateralis*
2	II	*Facies articularis antitrochanterica* midpoint	16–21	I	*Eminentia intercotylaris*
3–10	II	*Collum femoris circumference*	22–36	I	*Cotyla medialis*
12	I	*Trochanter femoris*	37	II	The most medial point of the lateral *Trochleae metatarsi IV*
13–20	I	*Crista trochanteris*	38	II	The most medial point of the medial *Trochleae metatarsi IV*
20–21	I	*Impressiones iliotrochanteris*	39	II	The most medial point of the lateral *Trochleae metatarsi III*
21–35	II	Medial edge of *femur*	40	II	The most medial point of the medial *Trochleae metatarsi III*
36–50	II	Lateral edge of *femur*	41	II	The most medial point of the lateral *Trochleae metatarsi II*
51–60	I	*Condylus medialis*	42	II	The most medial point of the medial *Trochleae metatarsi II*
61–70	I	*Sulcus intercondylaris*	43	I	*Eminentia intertrochlearis lateralis*
70–80	I	*Condylus lateralis*	44	I	*Eminentia intertrochlearis medialis*
***Tibiotarsus***			45–59	I	*Crista plantares lateralis*
1–8	I	*Facies articularis lateralis*	60–74	I	*Crista plantares medialis*
9–14	I	*Facies articularis medialis*			
15–22	I	*Crista patellaris*			
23–28	I	*Crista cnemialis cranialis*			
29–43	II	Medial edge of *tibiotarsus*			
44–58	II	Lateral edge of *tibiotarsus*			
59–68	I	*Condylus medialis*			
69–78	I	*Incisura intercondylaris*			
79–88	I	*Condylus lateralis*			
89	I	*Canalis extensorius*			

**Table 3:** Effect of different enzyme supplementation at different levels on performance parameters and European Production Efficiency Factor (EPEF) in broilers.

Period (day)	Groups						SEM	*p*-Value	Two-Way ANOVA (*p*-Value)		
	C	X100	X200	P250	P500	XP			XYL	PRO	XYL × PRO
	**Body weight (g)**										
1	45.27	45.42	45.32	45.39	45.18	45.32	0.040	0.583			
42	3395 ^b^	3589 ^a^	3543 ^a^	3531 ^ab^	3514 ^ab^	3585 ^a^	16.888	0.004	0.003	0.088	0.048
	**Body weight gain (g/period)**										
1–21	946.51	994.78	981.71	991.22	991.27	996.65	5.368	0.051			
22–42	2403 ^b^	2549 ^a^	2516 ^ab^	2495 ^ab^	2478 ^ab^	2543 ^a^	13.217	0.008	0.003	0.217	0.085
1–42	3350 ^b^	3543 ^a^	3498 ^a^	3486 ^ab^	3469 ^ab^	3540 ^a^	16.886	0.004	0.003	0.088	0.049
	**Feed intake (g/period)**										
1–21	1277 ^bc^	1291 ^abc^	1265 ^c^	1313 ^ab^	1295 ^abc^	1328 ^a^	5.405	0.003	0.161	0.007	0.966
22–42	3902 ^bc^	3984 ^a^	3963 ^ab^	3817 ^d^	3853 ^cd^	3880 ^cd^	11.915	<0.001	0.002	0.048	0.707
1–42	5179 ^abc^	5275 ^a^	5229 ^ab^	5131 ^d^	5147 ^cd^	5209 ^abc^	11.824	0.001	0.001	<0.001	0.615
	**Feed conversion ratio (kg feed/kg body weight gain-period)**										
1–21	1.350 ^a^	1.298 ^ab^	1.289 ^b^	1.327 ^ab^	1.306 ^ab^	1.333 ^ab^	0.006	0.040	0.048	0.187	0.047
22–42	1.625 ^a^	1.564 ^b^	1.576 ^ab^	1.530 ^b^	1.556 ^b^	1.527 ^b^	0.008	<0.001	0.052	<0.001	0.048
1–42	1.547 ^a^	1.489 ^b^	1.495 ^b^	1.472 ^b^	1.484 ^b^	1.472 ^b^	0.006	0.001	0.020	0.001	0.018
	**EPEF (%)**										
1–42	523.12 ^b^	574.11 ^a^	564.62 ^a^	571.54 ^a^	563.87 ^a^	580.22 ^a^	4.765	0.003	0.007	0.012	0.033

*n*: 6; abcd: values within the same row followed by different superscripts are significantly different (*p* < 0.05); SEM: standard error of mean; C: control; X100: 100 g/t xylanase (2.2 × 10^6^ U/t); X200: 200 g/t xylanase (4.4 × 10^6^ U/t); P250: 250 g/t protease (4.63 × 10^6^ U/t); P500: 500 g/t protease (9.25 × 10^6^ U/t); XP: 100 g/t xylanase (2.2 × 10^6^ U/t) + 250 g/t protease (4.63 × 10^6^ U/t); XYL: xylanase; PRO: protease; EPEF: European production efficiency factor.

**Table 4:** Effect of different enzyme supplementation at different levels on carcass yield (%) and relative internal organ weights (%) in broilers.

Items	Groups						SEM	*p*-Value
	C	X100	X200	P250	P500	XP		
Carcass	74.71	74.80	75.39	75.06	75.22	74.78	0.155	0.770
Liver	1.867	1.805	1.780	1.813	1.810	1.805	0.018	0.827
Heart	0.393	0.397	0.364	0.396	0.401	0.396	0.047	0.234
Spleen	0.080	0.075	0.078	0.088	0.084	0.084	0.002	0.371
Bursa of Fabricius	0.070	0.053	0.050	0.064	0.055	0.052	0.003	0.110
Gizzard	1.187	1.089	1.089	1.171	1.118	1.184	0.015	0.156
Proventriculus	0.263	0.257	0.261	0.254	0.254	0.281	0.003	0.094
Abdominal fat	1.078	1.097	0.985	1.208	1.010	1.185	0.035	0.346

*n*: 12; SEM: standard error of mean; C: control; X100: 100 g/t xylanase (2.2 × 10^6^ U/t); X200: 200 g/t xylanase (4.4 × 10^6^ U/t); P250: 250 g/t protease (4.63 × 10^6^ U/t); P500: 500 g/t protease (9.25 × 10^6^ U/t); XP: 100 g/t xylanase (2.2 × 10^6^ U/t) + 250 g/t protease (4.63 × 10^6^ U/t).

**Table 5:** Effect of different enzyme supplementation at different levels on intestinal viscosity (cP) in broilers.

Items	Groups						SEM	*p*-Value	Two-Way ANOVA (*p*-Value)		
	C	X100	X200	P250	P500	XP			XYL	PRO	XYL × PRO
Jejunum	1.463 ^a^	0.839 ^bc^	0.784 ^bc^	1.158 ^ab^	1.116 ^abc^	0.768 ^c^	0.047	<0.001	<0.001	0.034	0.208
Ileum	2.028 ^a^	1.478 ^b^	1.295 ^b^	1.595 ^b^	1.599 ^b^	1.259 ^b^	0.046	<0.001	<0.001	0.001	0.239

*n*: 12; abc: Values within the same row followed by different superscripts are significantly different (*p* < 0.05); SEM: standard error of mean; cP: centipoise; C: control; X100: 100 g/t xylanase (2.2 × 10^6^ U/t); X200: 200 g/t xylanase (4.4 × 10^6^ U/t); P250: 250 g/t protease (4.63 × 10^6^ U/t); P500: 500 g/t protease (9.25 × 10^6^ U/t); XP: 100 g/t xylanase (2.2 × 10^6^ U/t) + 250 g/t protease (4.63 × 10^6^ U/t); XYL: xylanase; PRO: protease.

**Table 6:** Effect of different enzyme types and inclusion levels on cecal volatile fatty acid composition in broilers.

Items	Groups						SEM	*p*-Value	Two-Way ANOVA (*p*-Value)		
	C	X100	X200	P250	P500	XP			XYL	PRO	XYL × PRO
	**Absolute concentration (mmol/g DM)**										
Acetic acid	0.816 ^c^	1.245 ^a^	0.921 ^b^	0.956 ^b^	0.992 ^b^	1.314 ^a^	0.025	<0.001	<0.001	0.001	0.320
Propionic acid	0.151 ^c^	0.285 ^a^	0.202 ^b^	0.211 ^b^	0.213 ^b^	0.308 ^a^	0.008	<0.001	<0.001	0.002	0.155
Isobutyric acid	0.010 ^b^	0.018 ^a^	0.017 ^a^	0.016 ^a^	0.016 ^a^	0.019 ^a^	0.001	<0.001	<0.001	0.006	0.092
Butyric acid	0.183 ^c^	0.389 ^a^	0.257 ^b^	0.263 ^b^	0.273 ^b^	0.423 ^a^	0.012	<0.001	<0.001	0.002	0.240
Isovaleric acid	0.008	0.016	0.013	0.014	0.014	0.016	0.009	0.239			
Valeric acid	0.012 ^d^	0.024 ^b^	0.015 ^cd^	0.019 ^c^	0.017 ^c^	0.029 ^a^	0.001	<0.001	<0.001	0.002	0.517
Isocaproic acid	0.002	0.002	0.001	0.002	0.002	0.001	0.0001	0.582			
Caproic acid	0.005	0.006	0.007	0.005	0.005	0.005	0.0003	0.398			
Total VFA	1.187 ^c^	1.985 ^a^	1.434 ^b^	1.485 ^b^	1.532 ^b^	2.116 ^a^	0.044	<0.001	<0.001	<0.001	0.135
Total BCFA	0.020 ^b^	0.036 ^a^	0.032 ^a^	0.031 ^a^	0.032 ^a^	0.037 ^a^	0.001	0.008	0.004	0.063	0.115
	**Relative proportion (% of total VFA)**										
Acetic acid	68.59 ^a^	62.75 ^b^	64.33 ^b^	64.39 ^b^	64.74 ^b^	62.37 ^b^	0.420	<0.001	<0.001	0.012	0.032
Propionic acid	12.68	14.31	14.15	14.14	13.92	14.42	0.204	0.143			
Isobutyric acid	0.864 ^b^	0.929 ^ab^	1.204 ^a^	1.036 ^ab^	1.060 ^ab^	0.927 ^ab^	0.037	0.103	0.022	0.385	0.331
Butyric acid	15.54 ^b^	19.60 ^a^	17.79 ^ab^	17.75 ^ab^	17.80 ^ab^	19.82 ^a^	0.382	0.013	0.003	0.217	0.258
Isovaleric acid	0.718	0.805	0.912	0.968	0.925	0.762	0.062	0.832			
Valeric acid	1.023 ^b^	1.227 ^ab^	1.042 ^b^	1.289 ^ab^	1.139 ^ab^	1.378 ^a^	0.040	0.049	0.229	0.088	0.540
Isocaproic acid	0.138 ^a^	0.083 ^ab^	0.097 ^b^	0.105 ^b^	0.096 ^b^	0.068 ^b^	0.004	<0.001	<0.001	0.002	0.317
Caproic acid	0.443 ^ab^	0.307 ^bc^	0.479 ^a^	0.320 ^bc^	0.321 ^bc^	0.253 ^c^	0.021	0.010	0.028	0.117	0.472
Total BCFA	1.721	1.818	2.213	2.109	2.081	1.757	0.086	0.431			
Total A/P	5.469 ^a^	4.462 ^b^	4.595 ^b^	4.622 ^b^	4.713 ^b^	4.400 ^b^	0.084	0.001	0.002	0.020	0.036

*n*: 12; abcd: Values within the same row followed by different superscripts are significantly different (*p* < 0.05); SEM: standard error of mean; C: control; X100: 100 g/t xylanase (2.2 × 10^6^ U/t); X200: 200 g/t xylanase (4.4 × 10^6^ U/t); P250: 250 g/t protease (4.63 × 10^6^ U/t); P500: 500 g/t protease (9.25 × 10^6^ U/t); XP: 100 g/t xylanase (2.2 × 10^6^ U/t) + 250 g/t protease (4.63 × 10^6^ U/t); XYL: xylanase; PRO: protease; DM: dry matter; VFA: volatile fatty acids; BCFA: branched chain fatty acids; A/P: acetate/propionate.

**Table 7:** Effects of different enzyme types and inclusion levels on cecal microflora (log_10_ CFU/g cecal digesta) in broilers.

Microorganisms	Groups						SEM	*p*-Value	Two-Way ANOVA (*p*-Value)		
	C	X100	X200	P250	P500	XP			XYL	PRO	XYL × PRO
Coliform bacteria	6.68 ^a^	5.94 ^b^	6.05 ^b^	6.21 ^ab^	6.16 ^ab^	5.77 ^b^	0.075	0.009	0.002	0.077	0.391
*Lactobacillus* spp.	7.15 ^c^	7.79 ^ab^	7.73 ^b^	7.89 ^ab^	8.12 ^ab^	8.43 ^a^	0.077	<0.001	0.001	<0.001	0.725

*n*: 12; abc,: values within a row with different superscripts differ significantly at *p* < 0.05; SEM: standard error of mean; C: control; X100: 100 g/t xylanase (2.2 × 10^6^ U/t); X200: 200 g/t xylanase (4.4 × 10^6^ U/t); P250: 250 g/t protease (4.63 × 10^6^ U/t); P500: 500 g/t protease (9.25 × 10^6^ U/t); XP: 100 g/t xylanase (2.2 × 10^6^ U/t) + 250 g/t protease (4.63 × 10^6^ U/t); XYL: xylanase; PRO: protease.

**Table 8:** Effect of different enzyme supplementation at different levels on intestinal histomorphological parameters in broilers.

Items	Groups						SEM	*p*-Value	Two-Way ANOVA (*p*-Value)		
	C	X100	X200	P250	P500	XP			XYL	PRO	XYL × PRO
	**Duodenum**										
Villus height, µm	1576 ^c^	1786 ^b^	1842 ^a^	1846 ^a^	1854 ^a^	1853 ^a^	12.291	<0.001	<0.001	<0.001	<0.001
Crypt depth, µm	110.23 ^d^	114.00 ^c^	123.93 ^a^	119.43 ^b^	126.00 ^a^	120.57 ^b^	0.717	<0.001	<0.001	<0.001	0.100
VH/CD	14.30 ^c^	15.67 ^a^	14.86 ^b^	15.47 ^a^	14.71 ^bc^	15.37 ^a^	0.074	<0.001	<0.001	0.002	<0.001
	**Jejunum**										
Villus height, µm	1168.66 ^c^	1221.38 ^ab^	1231.65 ^ab^	1210.18 ^b^	1215.98 ^ab^	1240.85 ^a^	3.681	<0.001	<0.001	<0.001	0.085
Crypt depth, µm	119.56 ^bc^	115.47 ^d^	121.17 ^ab^	117.30 ^cd^	123.38 ^a^	120.27 ^abc^	0.455	<0.001	0.003	<0.001	<0.001
VH/CD	9.78 ^c^	10.58 ^a^	10.17 ^b^	10.32 ^b^	9.86 ^c^	10.32 ^b^	0.041	<0.001	<0.001	0.004	<0.001
	**Ileum**										
Villus height, µm	868.87 ^c^	872.60 ^c^	906.19 ^b^	916.12 ^ab^	911.71 ^b^	935.35 ^a^	3.469	<0.001	<0.001	<0.001	0.136
Crypt depth, µm	128.80 ^b^	122.73 ^c^	132.03 ^ab^	130.97 ^ab^	134.03 ^a^	132.70 ^ab^	0.616	<0.001	<0.001	<0.001	0.001
VH/CD	6.76 ^c^	7.12 ^a^	6.87 ^bc^	7.00 ^abc^	6.80 ^c^	7.05 ^ab^	0.028	<0.001	0.002	0.198	0.011

*n*: 12; abcd: Values within the same row followed by different superscripts are significantly different (*p* < 0.05). SEM: standard error of mean; VH: villus height; CD: crypt depth; C: control; X100: 100 g/t xylanase (2.2 × 10^6^ U/t); X200: 200 g/t xylanase (4.4 × 10^6^ U/t); P250: 250 g/t protease (4.63 × 10^6^ U/t); P500: 500 g/t protease (9.25 × 10^6^ U/t); XP: 100 g/t xylanase (2.2 × 10^6^ U/t) + 250 g/t protease (4.63 × 10^6^ U/t); XYL: xylanase; PRO: protease.

**Table 9:** Effect of different enzyme supplementation at different levels on breast meat properties in broilers.

Items	Groups						SEM	*p*-Value
	C	X100	X200	P250	P500	XP		
WHC, %	20.70	19.37	19.09	20.09	20.81	19.91	0.254	0.293
Dry matter, %	24.72	25.05	25.11	24.89	24.84	24.75	0.107	0.881
Protein, %	22.38	22.74	22.73	22.67	22.61	22.48	0.099	0.888
Fat, %	1.16	1.15	1.17	1.05	1.08	1.14	0.030	0.857
Ash, %	1.18	1.17	1.21	1.17	1.15	1.14	0.009	0.344
TPC, mg GAE/g	0.96	0.97	1.01	1.01	1.09	0.97	0.018	0.321
DPPH RSA, %	69.07	69.33	70.45	69.20	69.37	71.75	0.343	0.167
TAS, mmol/kg	9.25	9.14	10.07	9.22	9.95	9.70	0.137	0.202
TOS, µmol/kg	13.41	10.85	13.82	13.12	13.95	13.70	0.567	0.636
OSI	0.147	0.121	0.137	0.145	0.140	0.143	0.006	0.864

*n*: 12; SEM: standard error of mean; WHC: Water-holding capacity; TPC: total phenolic content; GAE: gallic acid equivalent; DPPH: 2,2-diphenyl-picrylhydrazyl; RSA: radical scavenging activity; TAS: total antioxidant status; TOS: total oxidant status; OSI: oxidative stress index; C: control; X100: 100 g/t xylanase (2.2 × 10^6^ U/t); X200: 200 g/t xylanase (4.4 × 10^6^ U/t); P250: 250 g/t protease (4.63 × 10^6^ U/t); P500: 500 g/t protease (9.25 × 10^6^ U/t); XP: 100 g/t xylanase (2.2 × 10^6^ U/t) + 250 g/t protease (4.63 × 10^6^ U/t).

**Table 10:** Effect of different enzyme supplementation at different levels on the length, width, and thickness of *femur, tibiotarsus,* and *tarsometatarsus* in broilers.

Items, mm	Groups						SEM	*p*-Value	Two-Way ANOVA (*p*-Value)		
	C	X100	X200	P250	P500	XP			XYL	PRO	XYL × PRO
	*Femur*										
Total length	72.05 ^b^	74.98 ^a^	75.58 ^a^	73.40 ^ab^	75.08 ^a^	73.73 ^ab^	0.259	<0.001	<0.001	0.004	0.019
Proximal width	12.44 ^b^	13.06 ^ab^	14.23 ^a^	13.79 ^ab^	13.63 ^ab^	13.79 ^ab^	0.162	0.017	0.005	0.013	0.406
Distal width	16.24 ^b^	17.78 ^ab^	17.80 ^ab^	18.68 ^a^	18.48 ^a^	18.02 ^a^	0.181	0.001	0.146	0.001	0.006
Narrowest corpus width	4.33 ^c^	5.96 ^ab^	5.95 ^ab^	6.22 ^a^	6.42 ^a^	5.39 ^b^	0.106	<0.001	<0.001	<0.001	<0.001
Longest corpus width	10.37 ^c^	12.50 ^a^	11.80 ^ab^	12.32 ^ab^	11.67 ^b^	10.21 ^c^	0.129	<0.001	0.262	0.255	<0.001
Cranial corpus thickness	2.32 ^c^	2.83 ^ab^	2.25 ^c^	3.12 ^a^	2.98 ^a^	2.57 ^bc^	0.051	<0.001	0.009	0.001	<0.001
Caudal corpus thickness	2.27 ^c^	2.70 ^ab^	2.15 ^c^	2.95 ^a^	2.72 ^ab^	2.43 ^bc^	0.050	<0.001	0.015	0.070	<0.001
Lateral corpus thickness	2.00 ^bc^	2.42 ^ab^	1.63 ^c^	2.36 ^ab^	2.57 ^a^	1.70 ^c^	0.064	<0.001	0.001	0.016	<0.001
Medial corpus thickness	3.12 ^b^	2.84 ^c^	2.57 ^d^	3.51 ^a^	3.09 ^b^	3.24 ^b^	0.042	<0.001	<0.001	<0.001	0.923
	***Tibiotarsus***										
Total length	101.23	102.23	102.22	102.08	103.58	103.23	0.279	0.179			
Proximal width	24.45 ^ab^	24.27 ^b^	23.95 ^b^	25.26 ^ab^	25.87 ^a^	23.98 ^b^	0.164	0.001	0.085	0.066	0.127
Distal width	18.80 ^abc^	17.86 ^bc^	17.69 ^c^	19.48 ^a^	19.31 ^ab^	18.43 ^abc^	0.170	0.005	0.016	0.245	0.888
Narrowest corpus width	4.32 ^b^	3.70 ^c^	4.59 ^b^	4.37 ^b^	5.16 ^a^	4.45 ^b^	0.065	<0.001	<0.001	<0.001	0.001
Longest corpus width	9.96 ^c^	10.10 ^bc^	10.91 ^ab^	9.88 ^c^	10.44 ^abc^	11.26 ^a^	0.101	<0.001	<0.001	0.006	0.003
Cranial corpus thickness	2.65 ^b^	2.52 ^b^	2.40 ^b^	3.19 ^a^	2.53 ^b^	2.41 ^b^	0.044	<0.001	<0.001	<0.001	<0.001
Caudal corpus thickness	2.33 ^c^	2.82 ^ab^	2.82 ^ab^	2.99 ^a^	2.51 ^bc^	2.79 ^ab^	0.041	<0.001	0.014	<0.001	<0.001
Lateral corpus thickness	2.68 ^b^	3.23 ^a^	3.26 ^a^	3.27 ^a^	2.92 ^ab^	3.18 ^a^	0.048	<0.001	0.006	0.028	0.002
Medial corpus thickness	2.54 ^b^	3.57 ^a^	3.67 ^a^	3.80 ^a^	3.56 ^a^	3.60 ^a^	0.069	<0.001	<0.001	<0.001	<0.001
	***Tarsometatarsus***										
Total length	74.57 ^c^	77.99 ^a^	76.73 ^ab^	75.65 ^bc^	76.45 ^ab^	76.95 ^ab^	0.215	<0.001	<0.001	0.057	0.018
Proximal width	21.31 ^b^	22.49 ^a^	22.20 ^ab^	21.55 ^ab^	21.73 ^ab^	22.52 ^a^	0.110	0.001	<0.001	0.517	0.645
Distal width	18.70 ^ab^	19.14 ^a^	18.29 ^b^	18.80 ^ab^	19.03 ^a^	19.03 ^a^	0.071	0.004	0.001	0.389	0.506
Narrowest corpus width	3.63 ^c^	4.13 ^a^	3.45 ^c^	3.67 ^bc^	4.44 ^a^	4.09 ^ab^	0.058	<0.001	<0.001	<0.001	0.706
Longest corpus width	7.44 ^b^	7.95 ^b^	7.83 ^ab^	7.52 ^b^	8.06 ^b^	8.16 ^a^	0.049	<0.001	<0.001	<0.001	0.470
Cranial corpus thickness	1.72 ^c^	1.64 ^c^	2.01 ^b^	1.67 ^c^	1.99 ^b^	2.16 ^a^	0.027	<0.001	<0.001	<0.001	<0.001
Caudal corpus thickness	2.00 ^c^	1.97 ^c^	2.35 ^ab^	1.93 ^c^	2.11 ^bc^	2.47 ^a^	0.033	<0.001	<0.001	<0.001	<0.001
Lateral corpus thickness	2.12 ^cd^	1.98 ^d^	2.47 ^ab^	2.02 ^d^	2.29 ^bc^	2.61 ^a^	0.033	<0.001	<0.001	<0.001	<0.001
Medial corpus thickness	2.56 ^d^	3.04 ^c^	3.41 ^b^	2.51 ^d^	2.71 ^d^	3.77 ^a^	0.063	<0.001	<0.001	<0.001	<0.001

*n*: 12; abcd: values within the same row followed by different superscripts are significantly different (*p* < 0.05); SEM: standard error of mean; C: control; X100: 100 g/t xylanase (2.2 × 10^6^ U/t); X200: 200 g/t xylanase (4.4 × 10^6^ U/t); P250: 250 g/t protease (4.63 × 10^6^ U/t); P500: 500 g/t protease (9.25 × 10^6^ U/t); XP: 100 g/t xylanase (2.2 × 10^6^ U/t) + 250 g/t protease (4.63 × 10^6^ U/t); XYL: xylanase; PRO: protease.

**Table 11:** Principal component values of the shape analysed *femur*, *tibiotarsus*, and *tarsometatarsus*, %.

	*Femur*			*Tibiotarsus*			*Tarsometatarsus*		
	PC1	PC2	Both PCs	PC1	PC2	Both PCs	PC1	PC2	Both PCs
C-X100	22.3	15.6	37.9	38.2	16.0	54.2	20.7	16.9	37.6
C-X200	21.8	17.4	39.2	34.6	13.8	48.4	21.4	14.3	35.7
C-P250	31.7	13.7	45.4	30.5	18.3	48.8	17.3	12.6	29.9
C-P500	20.9	16.1	37.0	36.6	14.9	51.5	21.1	16.4	37.5
C-XP	21.6	17.7	39.3	26.5	16.4	41.9	20.0	17.1	37.1

C: control; X100: 100 g/t xylanase (2.2 × 10^6^ U/t); X200: 200 g/t xylanase (4.4 × 10^6^ U/t); P250: 250 g/t protease (4.63 × 10^6^ U/t); P500: 500 g/t protease (9.25 × 10^6^ U/t); XP: 100 g/t xylanase (2.2 × 10^6^ U/t) + 250 g/t protease (4.63 × 10^6^ U/t).

**Table 12:** Effect of different enzyme supplementation at different levels on weight, surface area, and volume of bones in broilers.

Items	Groups						SEM	*p*-Value	Two-Way ANOVA (*p*-Value)		
	C	X100	X200	P250	P500	XP			XYL	PRO	XYL × PRO
***Femur***											
Weight, g	18.61 ^d^	19.31 ^c^	20.69 ^b^	20.13 ^b^	21.66 ^a^	21.97 ^a^	0.157	<0.001	<0.001	<0.001	0.001
Surface area, mm^2^	5353	5258	5438	5480	5545	5597	53.232	0.454			
Volume, cm^3^	6.63	6.88	6.91	7.11	7.18	7.18	0.087	0.399			
***Tibiotarsus***											
Weight, g	26.02 ^d^	27.13 ^c^	28.97 ^b^	27.58 ^c^	29.61 ^b^	30.61 ^a^	0.198	<0.001	<0.001	<0.001	<0.001
Surface area, mm^2^	7811 ^a^	7077 ^b^	7175 ^ab^	6998 ^b^	7167 ^ab^	7063 ^b^	71.468	0.006	0.041	0.030	0.014
Volume, cm^3^	10.58	10.24	10.03	9.79	10.68	9.91	0.109	0.104			
***Tarsometatarsus***											
Weight, g	16.91 ^e^	18.00 ^d^	19.23 ^b^	18.64 ^c^	20.13 ^a^	20.68 ^a^	0.162	<0.001	<0.001	<0.001	0.001
Surface area, mm^2^	5189	4923	5061	5039	5257	5056	39.765	0.178			
Volume, cm^3^	6.63	6.62	6.60	6.63	6.74	6.61	0.044	0.953			

*n*: 12; abcde: values within the same row followed by different superscripts are significantly different (*p* < 0.05); SEM: standard error of mean; C: control; X100: 100 g/t xylanase (2.2 × 10^6^ U/t); X200: 200 g/t xylanase (4.4 × 10^6^ U/t); P250: 250 g/t protease (4.63 × 10^6^ U/t); P500: 500 g/t protease (9.25 × 10^6^ U/t); XP: 100 g/t xylanase (2.2 × 10^6^ U/t) + 250 g/t protease (4.63 × 10^6^ U/t); XYL: xylanase; PRO: protease.

## Data Availability

The data that support the findings of this study are available from the corresponding author upon reasonable request.

## Supplementary Materials

The following supporting information can be downloaded at: https://www.mdpi.com/article/10.3390/ani16030465/s1, Table S1: Statistical analyses related to the bones were obtained using PAST (v.4.03). Figure S1: Effect of different enzyme supplementation at different levels on performance parameters according to the periods (day) in broilers.

## Abbreviations

The following abbreviations are used in this manuscript:

AOAC	Association of Official Analytical Chemists
BCFA	Branched-chain fatty acid
BW	Body weight
BWG	Body weight gain
CT	Computed tomography
DPPH	2,2 diphenyl-1-picrylhydrazyl
EPEF	European production efficiency factor
FCR	Feed conversion ratio
FI	Feed intake
NSP	Non-starch polysaccharides
OSI	Oxidative stress index
PCA	Principal component analysis
TAS	Total antioxidant status
TOS	Total oxidant status
TPC	Total phenolic content
VFA	Volatile fatty acid
WHC	Water-holding capacity

## References

[1] Akyüz H.Ç., Onbaşılar E.E., Bayraktaroğlu A.G., Ceylan A. (2022). Age and sex related changes in fattening performance, dermatitis, intestinal histomorphology, and serum IgG level of slow- and fast-growing broilers under the intensive system. Trop. Anim. Health Prod..

[2] Akyüz H.Ç., Onbaşılar E.E. (2023). Carcass, visceral organ, and meat quality properties of two broiler hybrids differing in growth rates. Anim. Sci. J..

[3] Havenstein G.B., Ferket P.R., Qureshi M.A. (2003). Growth, livability, and feed conversion of 1957 versus 2001 broilers when fed representative 1957 and 2001 broiler diets. Poult. Sci..

[4] Zuidhof M.J., Schneider B.L., Carney V.L., Korver D.R., Robinson F.E. (2014). Growth, efficiency, and yield of commercial broilers from 1957, 1978, and 2005. Poult. Sci..

[5] Julian R.J. (2005). Production and growth related disorders and other metabolic diseases of poultry—A review. Vet. J..

[6] Gündoğar U.C., Onbaşılar E.E., Ahlat O. (2024). Effects of abrupt and gradual light/dark switching on growth performance, behavior, villus development, meat characteristics, and immunity of broilers. Anim. Sci. J..

[7] Oviedo-Rondón E., Wineland M., Funderburk S., Small J., Cutchin H., Mann M. (2009). Incubation conditions affect leg health in large, high-yield broilers. J. Appl. Poult. Res..

[8] Choct M. (2006). Enzymes for the feed industry: Past, present and future. World’s Poult. Sci. J..

[9] Cowieson A.J. (2010). Strategic selection of exogenous enzymes for corn/soy-based poultry diets. J. Poult. Sci..

[10] Erdaw M.M., Wu S., Iji P.A. (2017). Growth and physiological responses of broiler chickens to diets containing raw, full-fat soybean and supplemented with a high-impact microbial protease. Asian-Australas. J. Anim. Sci..

[11] Lee J., Oh H., Kim Y., Song D., An J., Chang S., Go Y., Cho H., Lee B., Kim W.K. (2023). Effects of exogenous protease on performance, economic evaluation, nutrient digestibility, fecal score, intestinal morphology, blood profile, carcass trait, and meat quality in broilers fed normal diets and diets considered with matrix value. Poult. Sci..

[12] Cowieson A., Bedford M. (2009). The effect of phytase and carbohydrase on ileal amino acid digestibility in monogastric diets: Complimentary mode of action?. World’s Poult. Sci. J..

[13] Longhini G., Qudsieh R., Lopes M., Silva I., Pais V., Netto R., Lovon M., Granghelli C., Faria D., Araujo L. (2025). Influence of xylanase inclusion on productive performance, egg quality and intestinal health of commercial laying hens fed energy-reduced diets. Animals.

[14] Wang T., Zhou N., Ding F., Hao Z., Galindo-Villegas J., Du Z., Su X., Zhang M. (2024). Xylanase enhances gut microbiota-derived butyrate to exert immune-protective effects in a histone deacetylase-dependent manner. Microbiome.

[15] Wang X., Li D., Xu Y., Ding X., Liang S., Xie L., Wang Y., Zhan X. (2024). Xylanase supplement enhances the growth performance of broiler by modulating serum metabolism, intestinal health, short-chain fatty acid composition, and microbiota. Animals.

[16] da Nobrega I.P.T., Teixeira L.D.V., Fascina V.B., Bittencourt L.C. (2025). Effect of protease supplementation in diets with or without copper sulfate and formaldehyde on the standardized digestibility of amino acids in broiler chickens. Animals.

[17] Siegert W., Zuber T., Sommerfeld V., Krieg J., Feuerstein D., Kurrle U., Rodehutscord M. (2019). Prececal amino acid digestibility and phytate degradation in broiler chickens when using different oilseed meals, phytase and protease supplements in the feed. Poult. Sci..

[18] Sanchez J., Thanabalan A., Khanal T., Patterson R., Slominski B.A., Kiarie E. (2019). Growth performance, gastrointestinal weight, microbial metabolites and apparent retention of components in broiler chickens fed up to 11% rice bran in a corn-soybean meal diet without or with a multi-enzyme supplement. Anim. Nutr..

[19] Olukosi O.A., Cowieson A.J., Adeola O. (2007). Age-related influence of a cocktail of xylanase, amylase, and protease or phytase individually or in combination in broilers. Poult. Sci..

[20] Walk C.L., Juntunen K., Paloheimo M., Ledoux D.R. (2019). Evaluation of novel protease enzymes on growth performance and nutrient digestibility of poultry: Enzyme dose response. Poult. Sci..

[21] Demiraslan Y., Demircioğlu İ., Güzel B.C. (2024). Geometric analysis of mandible using semilandmark in Hamdani and Awassi sheep. Ankara Univ. Vet. Fak. Derg..

[22] Onbaşılar E.E., Yalçın S., Bakıcı C., Batur B., Kartal Y.K., Ahlat O., Kılıçlı I.B., Yalçın S. (2025). Comprehensive evaluation of probiotic effects on laying hen physiology: From performance to bone and gut morphology. Animals.

[23] Dalga S., Aslan K. (2025). Principal component and discriminant function analysis of cranium and mandible in domestic buffalo (*Bos bubalis*). Ankara Univ. Vet. Fak. Derg..

[24] Fares M.A. (2025). Three-dimensional morphological variation and sexual dimorphism in the humerus of dromedary camels (*Camelus dromedarius*) from El Oued region: A geometric morphometric analysis. Ankara Univ. Vet. Fak. Derg..

[25] Aviagen (2019). Ross 308 Broiler Nutrition Specifications.

[26] AOAC (Association of Official Analytical Chemists) (2000). Official Methods of Analysis.

[27] King T., Sheridan R. (2019). Determination of 27 elements in animal feed by inductively coupled plasma-mass spectrometry. J. AOAC Int..

[28] Erol H., Yalçın S., Midilli M., Yalçın S. (2009). The effects of dietary glycerol on growth and laying performance, egg traits and some blood biochemical parameters in quails. Revue Méd. Vét..

[29] Onbaşılar I., Yalçın S., Gebes E.S., Yalçın S., Şahin A. (2023). Evaluation of modified dried vinasse as an alternative dietary protein source for broilers. Anim. Sci. J..

[30] Hashizawa Y., Kubota M., Kadowaki M., Fujimura S. (2013). Effect of dietary vitamin E on broiler meat qualities, color, water-holding capacity and shear force value, under heat stress conditions. Anim. Sci. J..

[31] Onbaşılar E.E., Gündoğar U.C., Çapar Akyüz H., Kartal Y.K., Yalçın S., Nemutlu E., Reçber T., Akyüz M.F., Onbaşılar D., Özkul B.Y. (2025). Impact of dietary shrimp waste on physical properties, chemical composition, amino acid profile, and antioxidant levels of breast meat. Vet. Sci..

[32] Shirazi O.U., Khattak M., Shukri N.A.M., Nasyriq M.N. (2014). Determination of total phenolic, flavonoid content and free radical scavenging activities of common herbs and spices. J. Pharmacogn. Phytochem..

[33] Jung S., Choe J.H., Kim B., Yun H., Kruk Z.A., Jo C. (2010). Effect of dietary mixture of gallic acid and linoleic acid on antioxidative potential and quality of breast meat from broilers. Meat Sci..

[34] Yalçın S., Gebeş E., Şahin A., Duyum H., Escribano F., Ceylan A. (2017). Sepiolite as a feed supplement for broilers. Appl. Clay Sci..

[35] Yalçınkaya H., Yalçın S., Ramay M.S., Onbaşılar E.E., Bakır B., Elibol F.K.E., Yalçın S., Shehata A.A., Basiouni S. (2024). Evaluation of Spirulina platensis as a feed additive in low-protein diets of broilers. Int. J. Mol. Sci..

[36] Yalçın S., Ramay M.S., Güntürkün O.B., Yalçın S.S., Ahlat O., Yalçın S., Özkaya M. (2023). Efficacy of mono- and multistrain synbiotics supplementation in modifying performance, caecal fermentation, intestinal health, meat and bone quality, and some blood biochemical indices in broilers. J. Anim. Physiol. Anim. Nutr..

[37] (2006). Microbiology of Food and Animal Feedingstuffs—Horizontal Method for the Enumeration of Coliforms—Colony-Count Technique.

[38] (1998). Microbiology of Food and Animal Feedingstuffs—Horizontal Method for the Enumeration of Mesophilic Lactic Acid Bacteria—Colony-Count Technique at 30 Degrees C.

[39] Fedorov A., Beichel R., Kalpathy-Cramer J., Finet J., Fillion-Robin J.-C., Pujol S., Bauer C., Jennings D., Fennessy F., Sonka M. (2012). 3D Slicer as an image computing platform for the Quantitative Imaging Network. Magn. Reson. Imaging.

[40] Bakıcı C., Kılıçlı İ.B., Yunus H.A., Ünal İ., Batur B. (2025). Evaluating sexual dimorphism in Romanov sheep: A comparative 3D shape analysis of manual and automated landmarking. Ann. Anat..

[41] Rolfe S., Pieper S., Porto A., Diamond K., Winchester J., Shan S., Kirveslahti H., Boyer D., Summers A., Maga A.M. (2021). SlicerMorph: An open and extensible platform to retrieve, visualize and analyse 3D morphology. Methods Ecol. Evol..

[42] Bookstein F.L. (1997). Landmark methods for forms without landmarks: Morphometrics of group differences in outline shape. Med. Image Anal..

[43] von den Driesch A. (1976). A Guide to the Measurement of Animal Bones from Archaeological Sites, as Developed by the Institut Für Palaeoanatomie, Domestikationsforschung Und Geschichte Der Tiermedizin of the University of Munich.

[44] Hammer Ø., Harper D.A. (2001). Past: Paleontological statistics software package for educaton and data anlysis. Palaeontol. Electron..

[45] Ravindran V. (2013). Feed enzymes: The science, practice, and metabolic realities. J. Appl. Poult. Res..

[46] Bedford M.R. (2018). The evolution and application of enzymes in the animal feed industry: The role of data interpretation. Br. Poult. Sci..

[47] Choct M., Hughes R.J., Wang J., Bedford M., Morgan A., Annison G. (1996). Increased small intestinal fermentation is partly responsible for the anti-nutritive activity of non-starch polysaccharides in chickens. Br. Poult. Sci..

[48] Cowieson A.J., Toghyani M., Kheravii S.K., Wu S.B., Romero L.F., Choct M. (2019). A mono-component microbial protease improves performance, net energy, and digestibility of amino acids and starch, and upregulates jejunal expression of genes responsible for peptide transport in broilers fed corn/wheat-based diets supplemented with xylanase and phytase. Poult. Sci..

[49] Angel C.R., Saylor W., Vieira S.L., Ward N. (2011). Effects of a monocomponent protease on performance and protein utilization in 7- to 22-day-old broiler chickens. Poult. Sci..

[50] Cowieson A.J., Masey O’Neill H.V. (2013). Effects of exogenous xylanase on performance, nutrient digestibility and caecal thermal profiles of broilers given wheat-based diets. Br. Poult. Sci..

[51] Freitas D., Vieira S., Angel C., Favero A., Maiorka A. (2011). Performance and nutrient utilization of broilers fed diets supplemented with a novel mono-component protease. J. Appl. Poult. Res..

[52] Cowieson A.J., Ravindran V. (2008). Effect of exogenous enzymes in maize-based diets varying in nutrient density for young broilers: Growth performance and digestibility of energy, minerals and amino acids. Br. Poult. Sci..

[53] Stefanello C., Dalmoro Y.K., Rosa D.P., Teixeira L., Sorbara J.-O.B., Cowieson A.J., Faruk M.U. (2024). Effects of dietary digestible amino acids and a novel exogenous sfericase protease on growth performance of broilers. Heliyon.

[54] Vieira S.L., De Freitas C.R., Horn R.M., Favero A., Kindlein L., Sorbara J.O.B., Umar-Faruk M. (2023). Growth performance and nutrient digestibility of broiler chickens as affected by a novel protease. Front. Anim. Sci..

[55] Tajudeen H., Hosseindoust A., Ha S., Moturi J., Mun J., Lee C., Park J., Lokhande A., Ingale S., Kim J. (2022). Effects of dietary level of crude protein and supplementation of protease on performance and gut morphology of broiler chickens. Eur. Poult. Sci..

[56] Ceylan N., Koca S., Golzar Adabi S. (2024). Effect of two different dietary endo-1, 4-β-xylanases on growth performance, intestinal histomorphology, caecal microbial population and short-chain fatty acid composition of broiler chickens. Ital. J. Anim. Sci..

[57] Hajati H., Rezaei M., Sayyahzadeh H. (2009). The effects of enzyme supplementation on performance, carcass characteristics and some blood parameters of broilers fed on corn-soybean meal-wheat diets. Int. J. Poult. Sci..

[58] Choct M., Hughes R.J., Bedford M.R. (1999). Effects of a xylanase on individual bird variation, starch digestion throughout the intestine, and ileal and caecal volatile fatty acid production in chickens fed wheat. Br. Poult. Sci..

[59] Nian F., Guo Y., Ru Y., Li F., Peron A. (2011). Effect of exogenous xylanase supplementation on the performance, net energy and gut microflora of broiler chickens fed wheat-based diets. Asian-Australas. J. Anim. Sci..

[60] Montagne L., Pluske J., Hampson D. (2003). A review of interactions between dietary fibre and the intestinal mucosa, and their consequences on digestive health in young non-ruminant animals. Anim. Feed Sci. Technol..

[61] Yuan J., Yao J., Yang F., Yang X., Wan X., Han J., Wang Y., Chen X., Liu Y., Zhou Z. (2008). Effects of supplementing different levels of a commercial enzyme complex on performance, nutrient availability, enzyme activity and gut morphology of broilers. Asian-Australas. J. Anim. Sci..

[62] Nabizadeh A., Golian A., Hassanabadi A., Zerehdaran S. (2017). Effects of nutrient density and exogenous enzymesin starter diet on performance, intestinal microflora, gut morphology and immune response of broiler chickens. Rev. Bras. De Ciência Avícola.

[63] Awad W.A., Ghareeb K., Abdel-Raheem S., Bohm J. (2009). Effects of dietary inclusion of probiotic and synbiotic on growth performance, organ weights, and intestinal histomorphology of broiler chickens. Poult. Sci..

[64] Singh A., Mandal R., Bedford M., Jha R. (2021). Xylanase improves growth performance, enhances cecal short-chain fatty acids production, and increases the relative abundance of fiber fermenting cecal microbiota in broilers. Anim. Feed Sci. Technol..

[65] Guilloteau P., Martin L., Eeckhaut V., Ducatelle R., Zabielski R., Van Immerseel F. (2010). From the gut to the peripheral tissues: The multiple effects of butyrate. Nutr. Res. Rev..

[66] Van Hoeck V., Wu D., Somers I., Wealleans A., Vasanthakumari B., Sanchez A.G., Morisset D. (2021). Xylanase impact beyond performance: A prebiotic approach in broiler chickens. J. Appl. Poult. Res..

[67] Masey-O’Neill H., Singh M., Cowieson A. (2014). Effects of exogenous xylanase on performance, nutrient digestibility, volatile fatty acid production and digestive tract thermal profiles of broilers fed on wheat-or maize-based diet. Br. Poult. Sci..

[68] Kiarie E., Romero L., Ravindran V. (2014). Growth performance, nutrient utilization, and digesta characteristics in broiler chickens fed corn or wheat diets without or with supplemental xylanase. Poult. Sci..

[69] Barekatain M.R., Choct M., Iji P.A. (2013). Xylanase supplementation improves the nutritive value of diets containing high levels of sorghum distillers’ dried grains with solubles for broiler chickens. J. Sci. Food Agric..

[70] Blaak E.E., Canfora E.E., Theis S., Frost G., Groen A.K., Mithieux G., Nauta A., Scott K., Stahl B., van Harsselaar J. (2020). Short chain fatty acids in human gut and metabolic health. Benef. Microbes.

[71] Den Besten G., Van Eunen K., Groen A.K., Venema K., Reijngoud D.-J., Bakker B.M. (2013). The role of short-chain fatty acids in the interplay between diet, gut microbiota, and host energy metabolism. J. Lipid Res..

[72] Morrison D.J., Preston T. (2016). Formation of short chain fatty acids by the gut microbiota and their impact on human metabolism. Gut Microbes.

[73] Moughan P.J., Ravindran V., Sorbara J.O. (2014). Dietary protein and amino acids-consideration of the undigestible fraction. Poult. Sci..

[74] Pieper R., Kroger S., Richter J.F., Wang J., Martin L., Bindelle J., Htoo J.K., von Smolinski D., Vahjen W., Zentek J. (2012). Fermentable fiber ameliorates fermentable protein-induced changes in microbial ecology, but not the mucosal response, in the colon of piglets. J. Nutr..

[75] Selim N.A., Waly A.H., Abdel Magied H.A., Habib H.H., Fadl A., Shalash S. (2015). Further benefits of xylanase enzyme supplementation to low energy corn-soybean meal broiler diets. Egypt. J. Nutr. Feed..

[76] Kim D.Y., Kim K.H., Lee E.C., Oh J.K., Park M.A., Kil D.Y. (2025). Effect of dietary supplementation of xylanase alone or combination of xylanase and beta-glucanase on growth performance, meat quality, intestinal measurements, and nutrient utilization in broiler chickens. Anim. Biosci..

[77] Skrivan M., Englmaierova M., Marounek M., Taubner T., Lanzoni D., Bejckova K., Giromini C., Baldi A. (2024). Dietary supplementation with xylanase suppresses the antinutritional effect of nonstarch polysaccharides of flaxseed and increases bone strength in broiler chickens. PLoS ONE.

[78] Petry A.L., Huntley N.F., Bedford M.R., Patience J.F. (2020). Xylanase increased the energetic contribution of fiber and improved the oxidative status, gut barrier integrity, and growth performance of growing pigs fed insoluble corn-based fiber. J. Anim. Sci..

[79] Pirgozliev V.R., Mansbridge S.C., Whiting I.M., Abdulla J.M., Rose S.P., Kljak K., Johnson A., Drijfhout F., Atanasov A.G. (2023). The benefits of exogenous xylanase in wheat–soy based broiler chicken diets, consisting of different soluble non-starch polysaccharides content. Poultry.

[80] Inayah S.R., Mutia R., Jayanegara A., Yanza Y.R., Amnah S. (2022). Effects of xylanase supplementation on the performance, nutrient digestibility, and digestive organ profiles of broiler chickens: A Meta-analysis. J. World’s Poult. Res..

[81] Maniraguha V., Hong J.S., Yu M., Oketch E.O., Yi Y.-J., Yun H., Jayasena D.D., Heo J.M. (2024). Feeding dietary non-starch polysaccharides supplemented with xylanase could improve the performance of broilers. J. Anim. Sci. Technol.

[82] Farquharson C., Jefferies D. (2000). Chondrocytes and longitudinal bone growth: The development of tibial dyschondroplasia. Poult. Sci..

[83] Hafeez A., Iqbal S., Sikandar A., Din S., Khan I., Ashraf S., Khan R.U., Tufarelli V., Laudadio V. (2021). Feeding of phytobiotics and exogenous protease in broilers: Comparative effect on nutrient digestibility, bone strength and gut morphology. Agriculture.

[84] Klingenberg C.P. (2016). Size, shape, and form: Concepts of allometry in geometric morphometrics. Dev. Genes Evol..

[85] Muszynski S., Arczewska M., Swiatkiewicz S., Arczewska-Wlosek A., Dobrowolski P., Swietlicka I., Hulas-Stasiak M., Blicharski T., Donaldson J., Schwarz T. (2020). The effect of dietary rye inclusion and xylanase supplementation on structural organization of bone constitutive phases in laying hens fed a wheat-corn diet. Animals.

[86] Cowieson A.J., Roos F.F. (2016). Toward optimal value creation through the application of exogenous mono-component protease in the diets of non-ruminants. Anim. Feed Sci. Technol..

[87] Farrokhi H., Abdullahpour R., Rezaeipour V. (2021). Influence of dietary phytase and protease, individually or in combination, on growth performance, intestinal morphology, microbiota composition and nutrient utilisation in broiler chickens fed sesame meal-based diets. Ital. J. Anim. Sci..

[88] Olukosi O., Beeson L., Englyst K., Romero L. (2015). Effects of exogenous proteases without or with carbohydrases on nutrient digestibility and disappearance of non-starch polysaccharides in broiler chickens. Poult. Sci..

[89] Kies A.K., De Jonge L.H., Kemme P.A., Jongbloed A.W. (2006). Interaction between protein, phytate, and microbial phytase. *In vitro* studies. J. Agric. Food Chem..

