# Supplementary material for: Effects of Xylanase and Protease Supplementation on Growth Performance, Meat Quality, Gut Health, Cecal Fermentation, and Bone Traits in Broiler Chickens
Source: Animals (Basel). 2026 Feb 2;16(3):465. doi: 10.3390/ani16030465 (PMC12897446; doi:10.3390/ani16030465)
Supplement: Supplementary file 1 [file animals-16-00465-s001.zip › animals-4084309-supplementary.pdf]

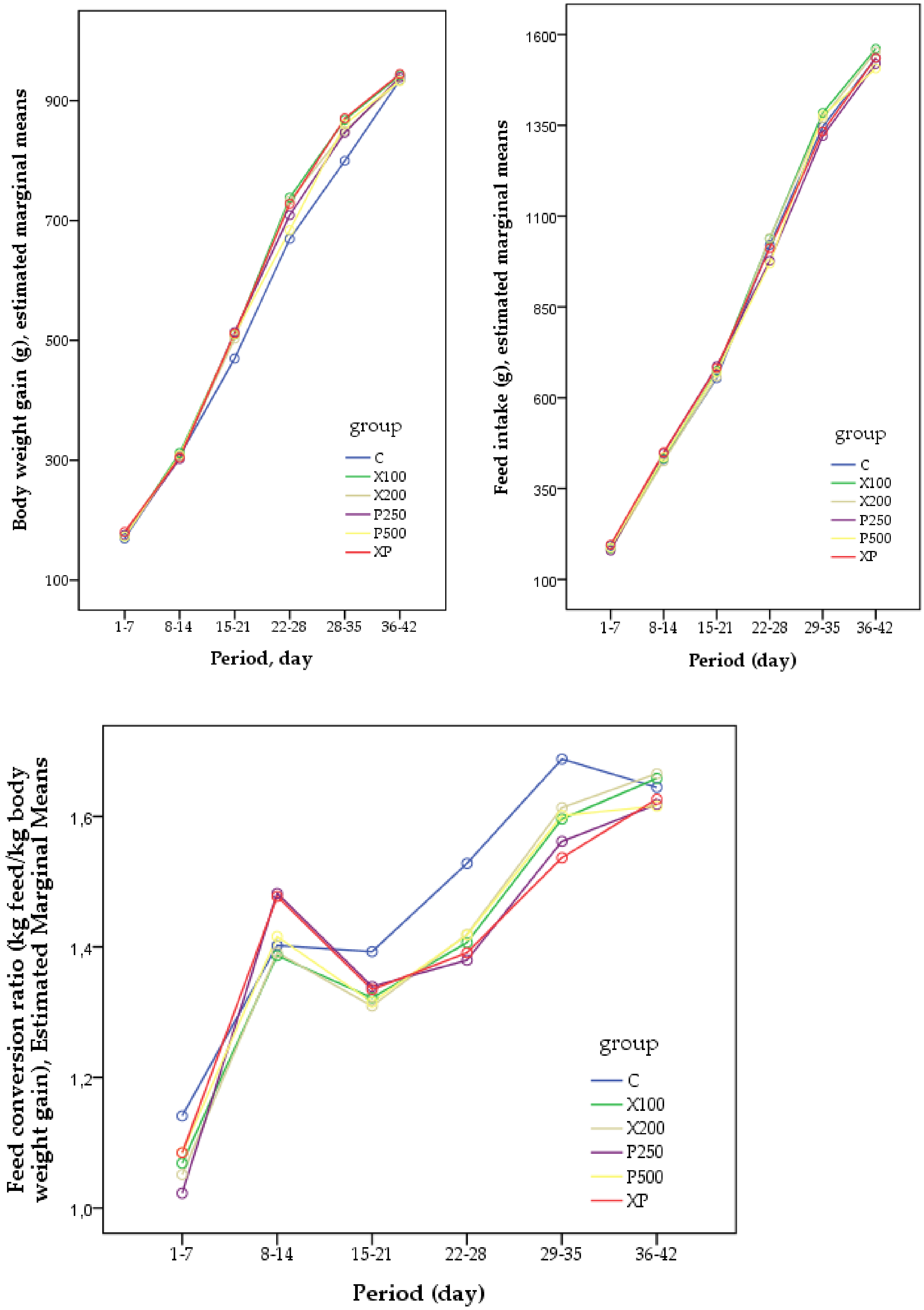

Figure S1. Effect of different enzyme supplementation at different levels on performance parameters according to the periods (day) in broilers. C: control; X100: 100 g/t xylanase (2.2 x 10^6 U/t); X200: 200 g/t xylanase (4.4 x 10^6 U/t); P250: 250 g/t protease (4.63 x 10^6 U/t); P500: 500 g/t protease (9.25 x 10^6 U/t); XP: 100 g/t xylanase (2.2 x 10^6 U/t) + 250 g/t protease (4.63 x 10^6 U/t).

Table S1 Statistical analyses related to the bones were obtained using PAST (v.4.03).

Groups	Mean Square Between Groups	F-value	*p*-Value	Levene´s test *p*-value	Shapiro Wilk-W	Shapiro Wilk *p*-value	Permutation *p* (n=99999)
	Femur						
C-X100	0.001	3.33	> 0.05	> 0.05	0.93	> 0.05	> 0.05
C-X200	0.001	7.19	< 0.05*	> 0.05	0.91	> 0.05	< 0.05*
C-P250	0.009	26.4	< 0.001*	> 0.05	0.82	< 0.05*	< 0.001*
C-P500	0.001	2.70	> 0.05	> 0.05	0.92	> 0.05	> 0.05
C-XP	0.003	23.83	< 0.001*	> 0.05	0.97	> 0.05	< 0.001*
	Tibiotarsus						
C-X100	0.016	51.0	< 0.001*	> 0.05	0.89	> 0.05	< 0.001*
C-X200	0.011	29.52	< 0.001*	> 0.05	0.95	> 0.05	< 0.001*
C-P250	0.001	3.47	> 0.05	< 0.05*	0.79	< 0.05*	> 0.05
C-P500	0.007	22.33	< 0.001*	< 0.05*	0.92	< 0.05*	< 0.001*
C-XP	0.003	8.59	< 0.001*	> 0.05	0.90	< 0.05*	< 0.001*
	Tarsometatarsus						
C-X100	0.001	13.43	< 0.001*	> 0.05	0.87	< 0.05*	< 0.001*
C-X200	0.001	3.17	> 0.05	> 0.05	0.72	> 0.05	> 0.05
C-P250	0.001	3.57	> 0.05	> 0.05	0.96	> 0.05	> 0.05
C-P500	0.001	3.37	> 0.05	> 0.05	0.76	> 0.05	> 0.05
C-XP	0.004	32.09	< 0.001*	> 0.05	0.92	> 0.05	< 0.001*

*Statistically significant, C: control; X100: 100 g/t xylanase (2.2 x 10^6 U/t); X200: 200 g/t xylanase (4.4 x 10^6 U/t); P250: 250 g/t protease (4.63 x 10^6 U/t); P500: 500 g/t protease (9.25 x 10^6 U/t); XP: 100 g/t xylanase (2.2 x 10^6 U/t) + 250 g/t protease (4.63 x 10^6 U/t).
